# CD14 Blockade Modulates Macrophage-Mediated Immunological Injury in a Translational Model of Reperfused ST-Segment Elevation Myocardial Infarction

**DOI:** 10.1016/j.jacbts.2025.101393

**Published:** 2025-10-23

**Authors:** Aascha A. D’Elia (nee Brown), Helen Kiriazis, Jason Bloom, Jonathan Noonan, Ian Hsu, Gabriella E. Farrugia, Haoyun Fang, Stephanie Jansen, Natalia Carvajal, Crisdion Krstevski, Waled A. Shihata, Yow Keat Tham, Angela Vais, Camilla Cohen, Adam C. Parslow, Chad Johnson, Anita C. Thomas, Malathi S.I. Dona, Kyah Grigolon, Scott J.Y. Loh, Guy Krippner, David K. Wright, Bing H. Wang, Antonio Abbate, Junedh Amrute, Kory Lavine, Mark W. Appleby, David Crowe, Garry Redlich, Brian W. Ziegelaar, Julie R. McMullen, David W. Greening, Alexander R. Pinto, David M. Kaye, Daniel G. Donner

**Affiliations:** a Translational Cardiology Centre, Baker Heart and Diabetes Institute, Melbourne, Australia; b Baker Department of Cardiometabolic Health, University of Melbourne, Melbourne, Australia; c Heart Failure Laboratory, Baker Heart and Diabetes Institute, Melbourne, Australia; d Department of Cardiology, Alfred Hospital, Melbourne, Australia; e Atherothrombosis and Vascular Laboratory, Baker Heart and Diabetes Institute, Melbourne, Australia; f School of Translational Medicine, Monash University, Melbourne, Australia; g Cardiac Cellular Systems Laboratory, Baker Heart and Diabetes Institute, Melbourne, Australia; h Molecular Proteomics Laboratory, Baker Heart and Diabetes Institute, Melbourne, Australia; i Alfred Medical Research and Education Precinct Animal Services, Melbourne, Australia; j Monash Sequencing, Monash University, Melbourne, Australia; k Cardiac Hypertrophy Laboratory, Baker Heart and Diabetes Institute, Melbourne, Australia; l Baker Department of Cardiovascular Research, Translation and Implementation, La Trobe University, Melbourne, Victoria, Australia; m Monash Histology, Monash University, Melbourne, Australia; n Baker Institute Microscopy Platform, Baker Heart and Diabetes Institute, Melbourne, Australia; o Bioimaging Platform, La Trobe University, Melbourne, Australia; p Department of Neuroscience, Monash University, Melbourne, Australia; q Biomarker Discovery Laboratory, Baker Heart and Diabetes Institute, Melbourne, Australia; r Robert M. Berne Cardiovascular Research Center, University of Virginia, Charlottesville, Virginia, USA; s Departments of Medicine, Pathology and Immunology, and Developmental Biology, Washington University School of Medicine, St Louis, Missouri, USA; t Implicit Bioscience Ltd, Brisbane, Australia and Seattle, Washington, USA; u Department of Physiology, Monash University, Melbourne, Australia

**Keywords:** antibody therapy, CD14, heart failure, immunomodulation, myocardial infarction

## Abstract

These preclinical trials provide the first evidence of cluster of differentiation 14 (CD14) blockade with a murine analogue of atibuclimab, a CD14-neutralizing antibody, preventing secondary immunological exacerbation of cardiac injury in a translational mouse model of reperfused ST-segment elevation myocardial infarction (STEMI), assessed using multiple clinical modalities. Multiomic studies suggest CD14 blockade downregulated macrophage-specific proinflammatory and tissue-wide remodeling processes without suppression of monocyte-macrophage infiltration or repair. These findings support a clinically practicable targeted immunomodulatory strategy of CD14 blockade initiated at reperfusion to prevent chronic immunological progression toward ischemic heart failure, and provide new insights into the pleiotropic roles of CD14 in inflammation and myocardial injury.

## Highlights

• This series of randomized and blinded preclinical trials is the first to investigate a clinically practicable CD14 blockade strategy initiated at reperfusion in a translational model of STEMI with key clinical features.
• Anti-CD14 antibody therapy with a murine analogue of atibuclimab mitigated post-acute progression of left ventricular dysfunction, dilatation and hemodynamic decompensation over 28 days, assessed by multiple translational modalities (including echocardiography, cardiac magnetic resonance, and invasive intracardiac pressure-volume catheterization).
• Integrative multiomic analyses revealed early targeted immunomodulation of the monocyte/macrophage without overt immunosuppression, proposing CD14 as a master regulator of the monocyte/macrophage response to acute myocardial injury.
• These data inform and support future fundamental and clinical research into CD14 blockade strategies, particularly in the setting of STEMI.

## Visual Abstract

**Figure:**
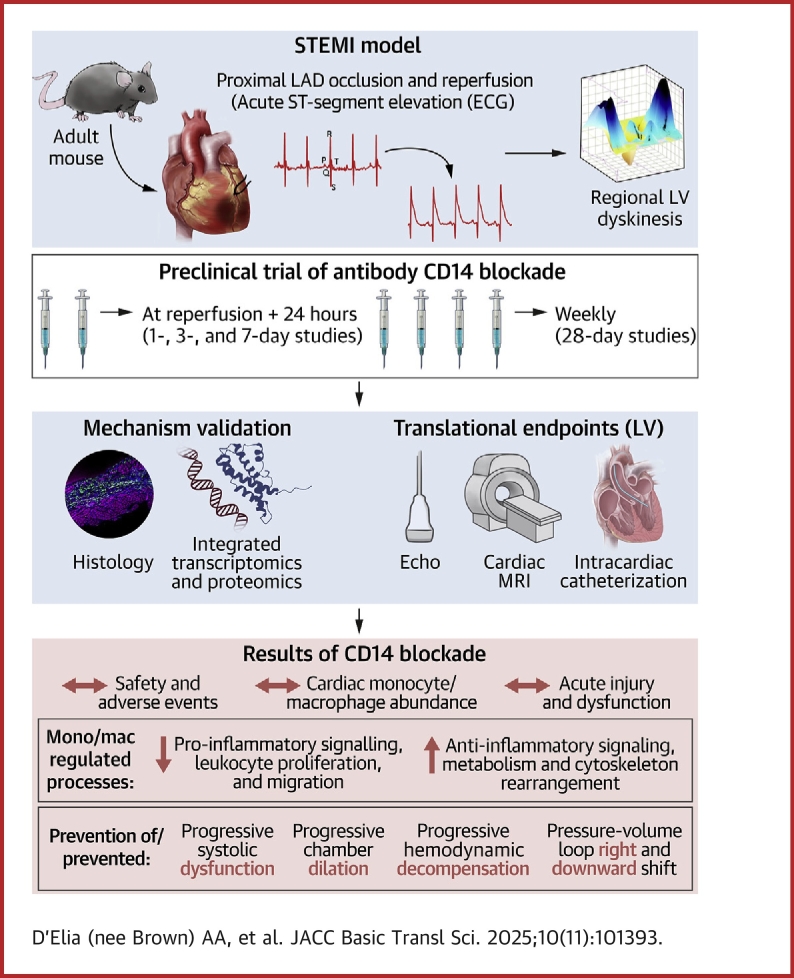

Ischemic heart disease is the leading cause of heart failure with reduced ejection fraction.^1^ Since the advent of reperfusion therapies and medical management of myocardial infarction (MI), mortality rates have steadily decreased whereas the number of patients with new-onset heart failure with reduced ejection fraction has not.^2^^,^^3^ ST-segment elevation myocardial infarction (STEMI) is a severe and life-threatening presentation of MI, associated with a total blockage/occlusion of 1 or more major coronary arteries causing transmural ischemia, myocardial damage, and progressive dysfunction and remodeling.^4^

Peripheral monocytosis and monocyte infiltration into the myocardium are hallmarks of post-MI inflammation,^5^ and are largely associated with poor clinical outcomes. Blood monocyte counts negatively correlate with ejection fraction (EF) post-MI^5^ in patients, and in preclinical murine MI models proinflammatory monocytes drive adverse remodeling by producing proinflammatory mediators and suppressing mechanisms of inflammatory resolution.^6^ Inhibiting the proinflammatory activities of monocytes and monocyte-derived macrophages following MI has been proposed as a key area for modern intervention.^7^, ^8^, ^9^ However, immunosuppression/monocyte depletion does not represent a viable therapeutic strategy. Experiments using broad anti-inflammatory drugs have yielded disappointing clinical outcomes, and were associated with destabilization of collagen deposition and inefficient clearance of dying cardiomyocytes (thus impacting healing and remodeling post MI).^8^^,^^10^, ^11^, ^12^, ^13^ These studies highlight the need to develop biologically informed strategies for selective immunomodulation of the monocyte-derived macrophage.^14^, ^15^, ^16^

Following MI, a maladaptive and excessive inflammatory response occurs, even with prompt and adequate reperfusion. Recent advancements have characterized the cell-specific contributors to secondary inflammatory damage and injury resolution following MI,^17^^,^^18^ identifying various distinct populations of monocytes and macrophages that exacerbate and resolve this damage according to their disparate and temporally modal phenotypes.^19^, ^20^, ^21^ During MI, the acquisition of a proinflammatory phenotype is influenced by Toll-like receptor signaling initiated by damage-associated molecular patterns (DAMPs) released from dying cells,^22^ resulting in consequent amplification of the innate and adaptive immune response through chemotactic and inflammatory secretions (such as debris phagocytosis, collagen deposition, and extracellular matrix stabilization).^23^

Cluster of differentiation 14 (CD14) is a widely validated marker for monocytes/macrophages, and has been proposed to be an upstream regulator of myeloid activity and a widely validated marker for systemic inflammation. CD14 is a coreceptor required for a wide array of DAMP signaling via Toll-like receptors by CD14^+^ pro-inflammatory monocytes and macrophages and release of cytokines like interleukin (IL)-1 and IL-6.^22^ Both soluble- and membrane-bound CD14 are implicated in adverse outcomes for patients following a range of cardiovascular diseases,^24^, ^25^, ^26^, ^27^ including heart failure.^28^ However, attempts to disrupt DAMP signaling through CD14 blockade are yet to be reported in the context of MI or adverse postischemic remodeling. An anti-CD14 antibody (Atibuclimab, Implicit Bioscience Ltd) has been studied as a targeted anti-inflammatory strategy in patients with amyotrophic lateral sclerosis (NCT04309604),^29^ arrhythmogenic cardiomyopathy (NCT06275893),^30^ and COVID-19 pneumonia (NCT04391309),^31^ showing a favorable safety profile, with clinical trials ongoing in arrhythmogenic cardiomyopathy and acute decompensated heart failure.

Here, we report the findings of the first experimental study of a novel anti-CD14 antibody, Atibuclimab analogue biG53 LALA-PG, in a translational murine model of reperfused STEMI with key clinical features, including acute and progressive left ventricular (LV) dysfunction and remodeling, respectively. These trials were designed to inform future clinical translation, and were subject to independent blinding, randomization, and state-of-the-art clinical modalities complemented in parallel by multi-omics technologies (single cell transcriptomics, cell- and tissue-based proteomics).

## Methods

All animal experimental work was performed in strict and routinely audited compliance with applications approved by the precinct’s Animal Ethics Committee (Projects: P1961 and P8295, Alfred Research Alliance, Melbourne, Australia). This study has been reported in line with the ARRIVE guidelines and in accordance with the National Institutes of Health Guide for the Care and Use of Laboratory Animals.

A total of 244 adult C57BL6 male mice (10 ± 1 weeks of age; AMREP AS) with STEMI were included in these studies. Multilevel randomization and blinding protocols were implemented across all studies in accordance with our center’s policy. Briefly, mice underwent STEMI surgery and were randomly allocated to groups receiving independently randomized, prepared, and blinded treatments (ie, saline, isotype, or anti-CD14). Accordingly, all procedures were performed, all samples/images/traces were collected and analyzed, all data were statistically compared, and each trial was individually reported to the trial sponsor while coded treatment blinding was maintained. An additional 10 age-matched sham-operated animals have been included in figures below where appropriate to provide reference values only, and are not included in our statistical analyses. Details are included in Table 1 and Supplemental Methods; for cohort sizes and adverse events see Supplemental Table S1.

**Table 1:** Predetermined Primary and Exploratory Endpoints

Assessment of Left Ventricle	Time Post-STEMI, d	Saline	Isotype	Anti-CD14	ANOVA *P* Value
Primary					
EF by echocardiography, %	7	25 ± 1	24 ± 2	31 ± 1^a^^,^^b^	<0.001
Exploratory					
Infarct size (% AAR) by EB/TTC	1	65 ± 4	66 ± 4	69 ± 3	0.750
EF by CMR, %	21	19 ± 1	20 ± 1	30 ± 1^a^^,^^b^	<0.001
EDV by CMR, μL	21	102 ± 8	107 ± 7	76 ± 5^a^^,^^b^	0.009
EF by echocardiography, %	28	24 ± 1	25 ± 2	30 ± 1^a^^,^^b^	<0.001
GLS by echocardiography, %	28	−6.8 ± 0.3	−6.6 ± 0.4	−8.5 ± 0.4^a^^,^^b^	<0.001
EDV by echocardiography, μL	28	111 ± 6	101 ± 5	86 ± 4^a^^,^^b^	<0.001
Developed pressure by PV cath, mm Hg	28	80 ± 2	80 ± 2	87 ± 2^a^^,^^b^	<0.001
+dP/dt by PV cath, mm Hg/s	28	6,962 ± 248	7,019 ± 263	8,023 ± 262^a^^,^^b^	0.010

Values are mean ± SEM. Statistical analyses were performed as analysis of variance (ANOVA) followed by Tukey’s post hoc testing to compare saline, isotype, and anti-CD14 groups.

AAR = area-at-risk; CMR = cardiac magnetic resonance imaging; dP/dt = change in pressure/change in time; EB/TTC = Evans blue/tri-tetrazolium chloride staining; EDV = end-diastolic volume; EF = ejection fraction; GLS = global longitudinal strain; PV cath = pressure-volume catheterization.

a Significant difference anti-CD14 vs saline.

b Significant difference anti-CD14 vs isotype. *P* < 0.05 was determined to be significant for multiple/pairwise comparisons.

A pilot study was first used to confirm an effective dose range (echocardiographic % EF) 7 days post-STEMI as the primary endpoint (Supplemental Methods, Supplemental Figures S1 and S2) before the main trials were initiated.

### Model of anterior STEMI with reperfusion

**Figure 1:**
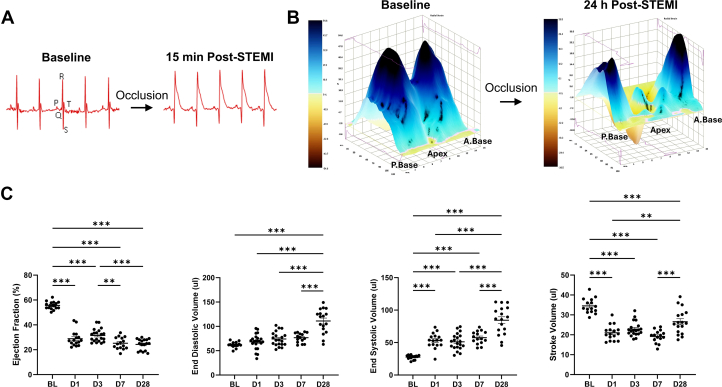
Characterization of the Mouse Model of Reperfused STEMI With Key Clinical Features Mice were given an anterior coronary artery occlusion, and reperfused 1 hour later. (A) Electrocardiograms were taken immediately after myocardial infarction surgery (ie, before reperfusion), demonstrating the distinct ST-segment elevation associated with occlusion of the left anterior coronary artery. (B) Representative 3-dimensional strain curves by echocardiography at baseline and 24 hours post–ST-segment elevation myocardial infarction (STEMI). (C) Echocardiographic assessment of left ventricular geometry and function from naïve and post-STEMI mice. Statistical analyses were performed using analysis of variance followed by Dunnett’s multiple comparison testing to compare baseline parameters with time elapsed after surgery. Mean ± SEM. ∗*P <* 0.05, ∗∗*P <* 0.01, ∗∗∗*P <* 0.001. A = anterior; BL = baseline; P = posterior.

We refined an existing preclinical model of reperfused MI with hallmarks specific to clinical STEMI, including classical ST-segment elevation, and progressive LV systolic dysfunction, dilatation, and hemodynamic decompensation. Mice underwent a surgical procedure to have the left anterior descending coronary artery reversibly ligated for 1 hour to induce ischemia followed by reperfusion, confirmed by classical ST-segment elevation by electrocardiogram (ECG) before each reperfusion (Figure 1A). Area-at-risk (AAR) was determined at 24 hours postsurgery using echocardiography (Figure 1B) (details can be found in Supplemental Methods and Supplemental Figure S3).

### LV structure and function

**Figure 2:**
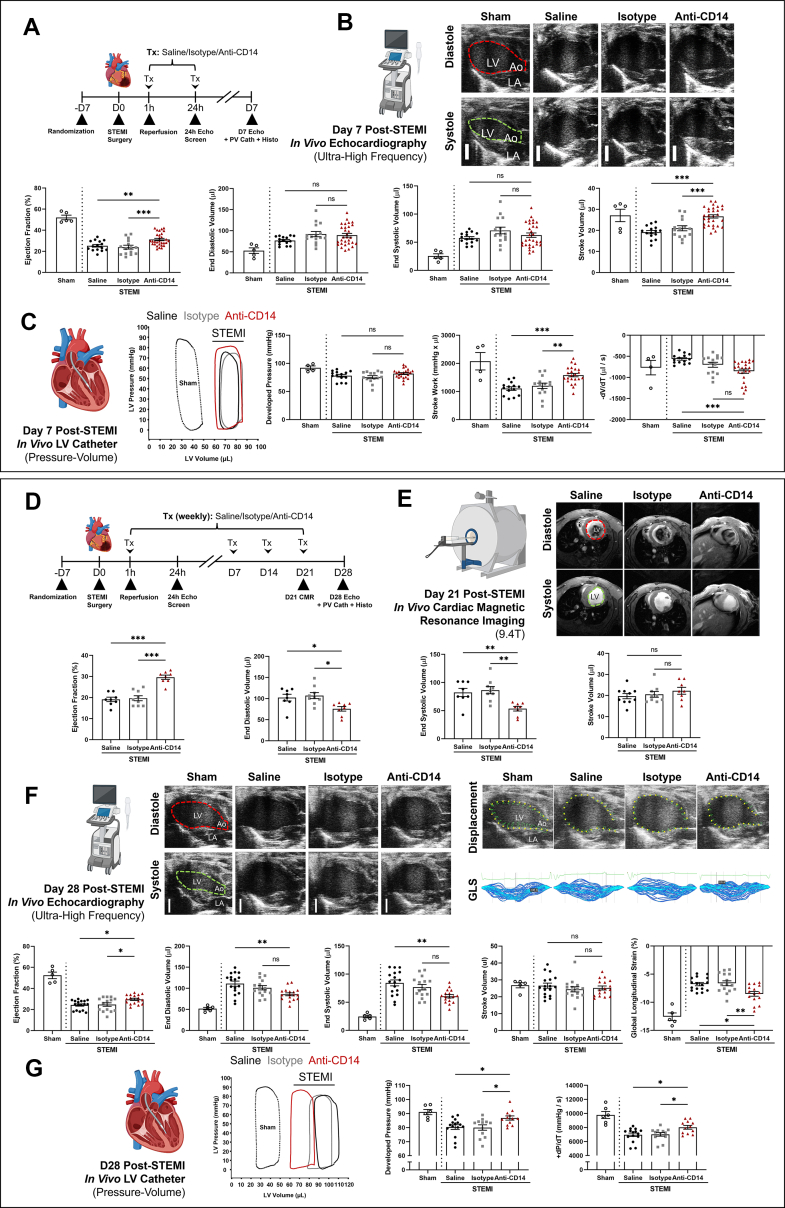
CD14 Blockade Preserves LV Systolic Function, Volumes, Biomechanics, and Hemodynamics 7 or 21 and 28 Days Post-STEMI After 1 hour left anterior coronary artery occlusion, mice were administered saline, IgG2a isotype control antibody, or anti-CD14 at the time of reperfusion (150 μL bolus, intravenous), with repeat injections at 24 hours (7-day experiment only) and weekly for up to 4 weeks. After 7 days, overt LV dysfunction, dilatation, and adverse hemodynamic changes were observed in saline- and isotype-treated mice, and these changes were significantly ameliorated with anti-CD14 treatment (Tx). Multimodality assessments of LV function and volumes by echocardiography, cardiac magnetic resonance imaging, and intracardiac pressure-volume hemodynamics revealed anti-CD14 Tx prevented progressive LV dysfunction and dilatation over 28 days post-STEMI. (Top) (A) 7-day trial design overview. (B) Echocardiographic assessment of LV geometry and function at 7 days post-STEMI. (C) Intracardiac catheter assessments of LV pressure-volume hemodynamics at 7 days post-STEMI. Single representative echocardiographic frames (parasternal long-axis view) (B), pressure-volume loops (C) are shown. (Bottom) (D) 28-day trial design overview. (E) Cardiac magnetic resonance imaging of LV volumes and function 21 days post-STEMI. (F) Echocardiographic assessments of LV volumes, systolic function, and biomechanics 28 days post-STEMI. (G) Intracardiac catheter assessments of LV pressure-volume hemodynamics. Single representative frames from CMR (E) and echocardiography (F, parasternal long-axis views, endocardial displacement, and global longitudinal strain [GLS]); and pressure-volume loops (G) are shown. Statistical analyses were performed using analysis of variance followed by Tukey’s post hoc testing to compare saline, isotype, and anti-CD14 groups only. Dotted lines and empty circles denote sham-operated controls provided for reference only, not included in statistical analyses. Mean ± SEM.∗*P <* 0.05, ∗∗*P <* 0.01, ∗∗∗*P <* 0.001. ns = not significant; other abbreviations as in Figure 1.

Parasternal long-axis echocardiography studies of LV volumes and systolic function were performed under isoflurane anesthesia using the Vevo 2100 system with a MS-550D transducer (VisualSonics, Fujifilm), operated by a senior imaging specialist at 1, 3, 7, and 28 days post-STEMI. All off-line analyses of ultra-high-frequency parasternal long-axis loops were critically performed by 2 independent observers using the manufacturer software (VevoLab version 5.0, VisualSonics, Fujifilm). Cardiac catheterization and hemodynamic assessments were performed within 24 hours of endpoint echocardiography at 7 and 28 days post-STEMI (for predetermined primary and exploratory endpoints see Table 1). Multislice (5-slice) short-axis cines were acquired by cardiac magnetic resonance (CMR) imaging in randomized subcohorts of mice from each group at 21 days post-STEMI (further details and examples can be found in Figure 2, Supplemental Methods, and Supplemental Figures S3 to S6. All echocardiographic, intracardiac catheter, and CMR imaging data are presented as mean ± SEM throughout results, figures and tables (unless otherwise stated).

### Mechanistic studies

Separate cohorts of animals were humanely killed at 1 or 3 days post-STEMI by anesthetic overdose and exsanguination. Plasma samples were used for cytokine assessment, while LV tissue was used for (immuno)histology, flow cytometry and cell sorting, single cell (macrophage-specific) RNA sequencing, and single-cell (macrophage-specific) and whole-tissue proteomics (further details and examples can be found in Supplemental Methods, Supplemental Tables S2 to S5, and Supplemental Figures S7 to S11.

### Statistical analysis

All biometric, physiological, histological, and cytometric data were analyzed using GraphPad Prism version 9.4.1 (GraphPad Software) using 1-way analysis of variance (ANOVA) with Tukey's (all pairwise) or Dunnett's (pairwise with baseline) post hoc test for multiple comparisons, as appropriate. Normality was assessed for all parameters using the Shapiro-Wilk test. Circulating cytokine and growth factor levels are presented as median with 25th to 75th percentiles (Q1-Q3) and compared using the Mann-Whitney *U* test (2 groups) or Kruskal-Wallis test (>2 groups). A *P* value <0.05 was determined to be statistically significant for all biometric, physiological, histological, and cytometric data sets. Gene Ontology (GO) enrichment analysis was conducted using the “enrichGO” function within the “clusterProfiler” R package version 4.4.4. Reference genomes obtained from GO were employed for all presented GO analyses. Enrichment analysis specifically for GO Biological Process (GO-BP) terms involved mapping our set of differentially expressed genes (adjusted *P* < 0.01) to the *Mus musculus* background gene list. Statistically significant GO-BP terms were identified using a Benjamini-Hochberg adjusted *P* value cutoff of 0.01. Proteome intensities were processed via VSN normalization method (Limma package version 3.65.3) in R (further details are available in Supplemental Methods).

## Results

### CD14 blockade with a novel murine antibody achieves high receptor occupancy without myeloid cell depletion

We have developed a nondepleting murine antimouse CD14 monoclonal antibody (biG53 LALA-PG monoclonal antibody [mAb] immunoglobulin G [IgG] 2a: see Supplemental Methods for details), described herein as anti-CD14, with 3 Fc region silencing mutations to cripple antibody-dependent cellular cytotoxicity, complement-dependent cytotoxicity, and antibody-dependent cellular phagocytosis function (Implicit Bioscience Ltd). Notably, these mutations were not available in a commercial IgG2a isotype control, limiting the broad interpretation of data relating to the isotype control used in these studies. We performed receptor occupancy studies to characterize the monocyte CD14 saturation of this mAb, and found that a single 5 mg/kg infusion of anti-CD14 achieved >80% mCD14 receptor occupancy on C57BL/6 mouse monocyte/macrophages (SSC lo, CD11b+, lymphocyte antigen 6 [Ly6] G−, CD115+ cells) for 21 days, and remained above baseline beyond that time. The same dose of anti-CD14 did not result in the depletion of CD14+ myeloid cells (measured over 29 days), and also inhibited lipopolysaccharide-dependent cytokine production in RAW264.7 cells (Supplemental Figure S1).

No adverse events (including signs of infection) were observed following treatment with anti-CD14 antibody either in vivo or at postmortem in any of the present studies (postmortem examinations performed for all animals, including all 102 mice following treatment with anti-CD14) (Supplemental Table S1).

### A newly characterized translational model of reperfused STEMI exhibits clinical hallmarks of systolic dysfunction and progressive remodeling

Here we sought to enhance the translatability of our trials by first refining an existing mouse model of reperfused STEMI characterized by key clinical features of LV ischemic injury, dysfunction, and progressive remodeling. All 204 animals randomized to STEMI treatment groups were confirmed to exhibit the following: 1) ST-segment elevation by ECG during occlusion of the proximal left anterior descending coronary artery, before reperfusion (Figure 1A); and 2) an AAR (as determined by echocardiographic endocardial displacement mapping assessed at 24 hours post-STEMI) involving between 35% and 55% of the longitudinal LV endocardium, inclusive (Figure 1B, Supplemental Figure S3). Any animals that did not meet these 2 criteria were excluded from the study.

This refined model of validated STEMI develops distinct phases of acute and persistent LV systolic dysfunction. At 1 and 3 days post-STEMI, acute systolic dysfunction was observed in the absence of overt dilatation. By D7, systolic dysfunction had progressively worsened. However, LV dilation developed after this timepoint, observed only at 28 days post-STEMI in these echocardiographic studies (Figure 1C) (end-diastolic volume: D28 111 ± 6.05 μL vs baseline 62.1 ± 1.50 μL, D1 68.3 ± 3.37 μL, D3 74.1 ± 3.24 μL, and D7 76.7 ± 2.54 μL; *P* < 0.001). Echocardiographic data for temporal model characterization was determined from saline-treated STEMI groups of the trials reported in the following text.

### Preclinical trials of CD14 blockade in reperfused STEMI

A series of in vivo trials investigating the effect of CD14 blockade on cardiac structure and function were performed, with endpoints assessed at 1, 3, 7, 21, and 28 days post-STEMI, and mechanistic studies (ie, cardiac cell sorting, single-cell RNA sequencing [scRNAseq], and proteomics) at earlier timepoints (1 and/or 3 days post-MI). Before the start of these experiments, a dosage pilot study (Supplemental Figure S2) was performed to inform the sufficient powering of the independent main studies for echocardiographic EF % at 7 days post-STEMI, alongside saline- and mouse IgG2a isotype-treated STEMI control groups. For full details of methods, see the Supplemental Methods.

#### CD14 blockade limits the progression of LV dysfunction and dilatation beyond 3 days post-STEMI

Parasternal long-axis 3-chamber echocardiography was performed and LVEF was assessed in separate cohorts of animals at 1, 3, 7, and 28 days post-STEMI (Figures 2A and 2B). These imaging studies revealed that anti-CD14 antibody treatment administered at reperfusion resulted in significantly greater LV systolic function at 7 days post-STEMI compared with saline- and isotype-treated control subjects (31% ± 1% vs 25% ± 1% and 24% ± 2% LVEF in saline- and isotype-treated groups, respectively; *P* < 0.001 primary outcome, confirmed power = 0.964 with alpha = 0.05). CD14 blockade also maintained physiological LV stroke volume (27 ± 1 μL vs 27 ± 3 μL in shams; *P* > 0.99) and cardiac output (14 ± 1 mL/min vs 14 ± 2 mL/min in shams; *P* > 0.99) at 7 days post-STEMI. At 1 and 3 days post-STEMI, no significant difference was observed between groups (Supplemental Figure S3).

Favorable treatment effects were also observed in pressure-volume (PV) hemodynamics at 7 days post-STEMI (Figure 2C), including greater stroke work (1,582 ± 62 mm Hg vs 1,103 ± 63 mm Hg and 1,201 ± 96 mm Hg × μL in saline- and isotype-treated groups, respectively; *P* < 0.01), and LV volume ejection rate (−852 ± 50 μL/s vs −563 ± 30 μL/s change in volume/change in time in saline-treated control subjects only; *P* < 0.001).

The beneficial effects of anti-CD14 treatment were associated with a ∼50% reduction in remote interstitial fibrosis at 7 days (0.6% ± 0.1% vs 1.2% ± 0.2%, and 1.1% ± 0.2%; *P* < 0.05) and 28 days post-STEMI (0.5% ± 0.1% vs 0.9% ± 0.1%, and 0.8% ± 0.1%; *P* < 0.01), and a modest ∼20% reduction in scar size (12% ± 1% vs 16% ± 2%, and 16% ± 1%; *P* < 0.01) at 7 days post-STEMI, compared with saline- and isotype-treated groups, respectively (Supplemental Figure S4), with no significant differences at 3 days (14 ± 2%, 15 ± 1%, and 15 ± 1% total LV, for saline, isotype, and anti-CD14-treated groups, respectively; *P* > 0.83).

In a separate experiment with clinical assessments at 21 and 28 days post-STEMI, multimodality, high spatial resolution assessment of LV structure and function was performed by either 9.4-T CMR or echocardiography, respectively (Table 1, Figures 2D to 2F). Multiplane short-axis CMR performed at 21 days post-STEMI demonstrated greater LVEF with anti-CD14 antibody treatment compared with both saline- and isotype-treated mice (30% ± 1% vs 19% ± 1% and 20 ± 1%, respectively; *P* < 0.001). CMR also revealed mitigated LV dilatation in anti-CD14-treated mice compared with control groups (76 ± 5 μL vs 102 ± 8 μL and 107 ± 7 μL end-diastolic volume in saline- and isotype-treated control subjects, respectively; *P* < 0.05) (Figure 2E).

In agreement with CMR at 21 days post-STEMI, parasternal long-axis echocardiography at 28 days post-STEMI confirmed that anti-CD14 treatment was associated with greater LV systolic function compared with both saline and isotype control subjects (30% ± 1% vs 24% ± 1%, and 25% ± 2% EF, respectively; *P* < 0.05) (Figure 2F). Similarly, preservation of LV end-diastolic (86 ± 4 μL vs 111 ± 6 μL, and 101 ± 5 μL; *P* < 0.01) and end-systolic (61 ± 3 μL vs 84 ± 5 μL, and 77 ± 5 μL; *P* < 0.01) volumes was also confirmed by echocardiography at 28 days post-STEMI in anti-CD14-treated mice (compared with the saline control group only) (Figure 2F). Additionally, global longitudinal strain (endocardial shortening; global longitudinal strain) of the LV was also significantly greater at 28 days post-STEMI with anti-CD14 treatment relative to both saline- and isotype-treated control subjects (−8.5% ± 0.4% vs −6.8% ± 0.3%, and −6.6% ± 0.4%, respectively; *P* < 0.05) (Figure 2F).

To determine whether the imaging findings were associated with changes in cardiovascular hemodynamics, invasive LV catheterization studies were also performed in vivo 28 days post-STEMI. Findings included preservation of LV developed pressure with anti-CD14 antibody treatment compared with saline- and isotype-treated control subjects (87 ± 2 mm Hg vs 80 ± 2 mm Hg, and 80 ± 2 mmHg; *P* < 0.05) (Figure 2G). Moreover, anti-CD14 antibody treatment resulted in greater positive change in pressure/change in time relative to both saline and isotype groups (8,023 ± 262 mm Hg/s vs 6,962 ± 248 mm Hg/s, and 7,019 ± 263 mmHg/s, respectively; *P* < 0.05) (Figure 2G).

Taken together, intracardiac hemodynamic examinations performed at 7 and 28 days post-STEMI revealed a progressive rightward shift of the PV loop in saline- and isotype-treated groups (Figures 2C and 2G) (indicating progressive LV dilatation). In contrast, loop position and morphology remained comparatively stationary with anti-CD14 treatment between these 2 timepoints.

#### CD14 blockade reprograms the cardiac macrophage phenotype following MI independent of infarct size

**Figure 3:**
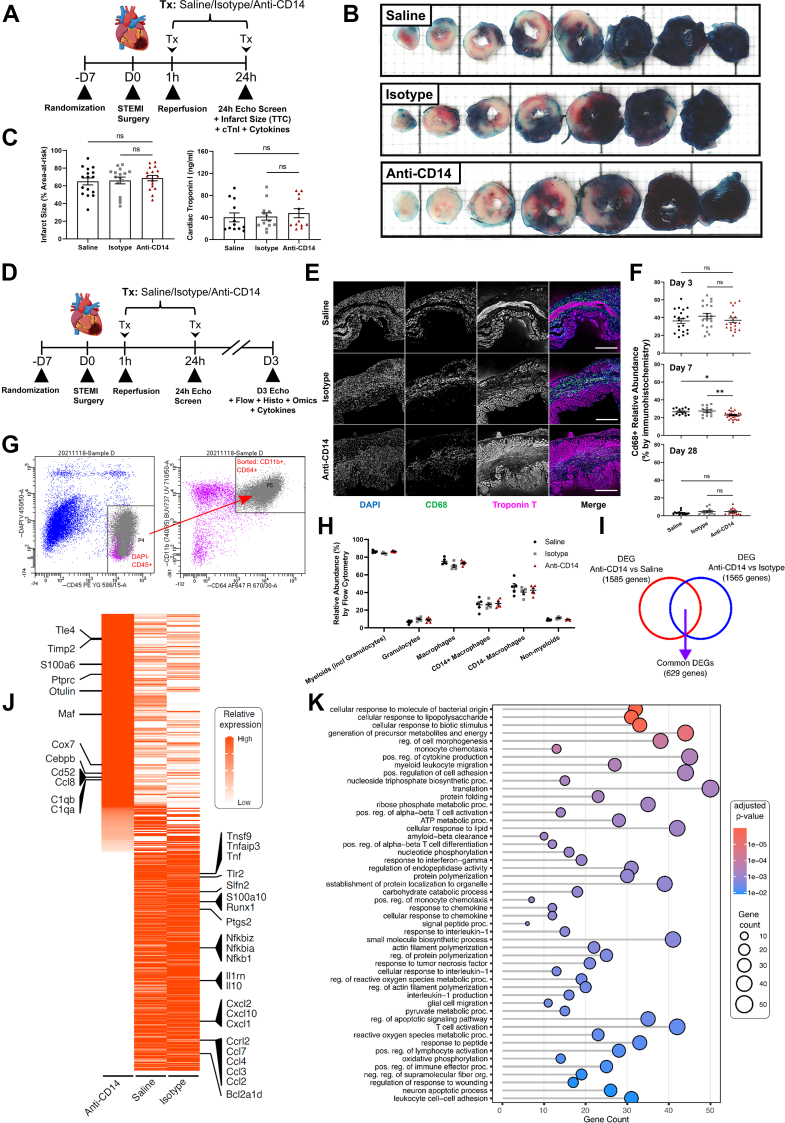
Acute Injury Assessment and Monocyte/Macrophage Abundance and Transcriptome Response to CD14 Blockade 1 and 3 Days Post-STEMI Acute infarct size and cardiac troponin I (a marker of cardiomyocyte death) were unaffected by anti-CD14 antibody treatment. At 3 days post-STEMI, macrophage abundance was similar between all groups. However, in these macrophages, widespread down-regulation of proinflammatory genes and up-regulation of anti-inflammatory genes were observed with anti-CD14 treatment. (A) Trial design overview for 24-hour acute injury assessment study. (B) Representative images of Evans blue/tri-tetrazolium chloride-stained hearts (grid 200 μm) are shown for each group. (C) Outcome measures of acute injury at 24-hour post-STEMI. (D) Trial design overview for 3-day macrophage abundance and phenotyping study. (E) Representative images of immunohistochemistry showing CD68+ cell accumulation within the infarcted myocardium (troponin T+ cardiomyocytes). (F) Immunohistochemistry analyses of macrophage (CD68+ cell) relative abundance at 3, 7, and 28 days post-STEMI. (G) Cell sorting protocol. Cells were isolated from the infarct region at 3 days post-STEMI, and populations identified using labelled antibodies. Live CD45^+^ CD11b^+^ CD64^+^ cells were used for pseudo-bulk RNA sequencing studies. (H) Flow cytometric readouts for relative abundance of leukocyte populations 3 days post-STEMI. (I) Identification of differentially expressed genes (DEG) common to anti-CD14 vs saline and anti-CD14 vs isotype comparisons. (J) Heat map of 629 common DEGs identified as described in (I). (K) Functional enrichment analysis of genes with decreased transcript levels in cardiac macrophages from the anti-CD14 treatment group. Mean ± SEM. ∗*P <* 0.05, ∗∗*P <* 0.01. Abbreviations as in Figures 1 and 2.

**Figure 4:**
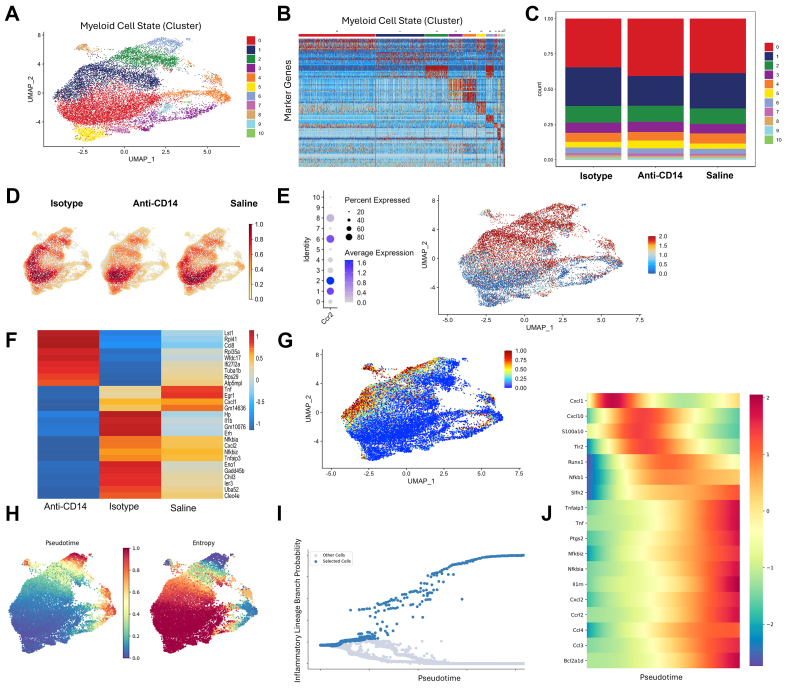
Single-Cell Analysis of Immune Cell Transcriptome Isolated From Infarcted Myocardium Following CD14 Blockade 3 Days Post-STEMI Anti-CD14 treatment blunts the monocyte proinflammatory lineage post-MI. Single-cell RNA sequencing was performed on sorted cells isolated from the infarct region at 3 days post-STEMI (described in Figure 3). (A) Uniform manifold approximation and projection (UMAP) plot displaying all 11 different populations of macrophages (>96%), dendritic cells (<3%), and neutrophils (<1%) identified in the apical region of the damaged heart at 3 days post-STEMI, and color chart (also applicable to B and C). (B) Differential expression marker genes for myeloid cell states. (C) Relative abundance histogram stack (cell composition plot), and (D) cell population density heat-map (gaussian distribution: 0-1) highlighting the reduced abundance of macrophages with high expression of Plac8/Ly6c2 (cluster 1; Ly6c2^hi^) in the anti-CD14 treatment group. (E) Marker expression dot-plot (including average scaled expression) and UMAP plot showing that *CCR2* was highly expressed in select subpopulations in the UMAP space (including cluster 1 and cluster 2), dendritic cells (cluster 6), and neutrophils (cluster 8), and poorly expressed in cells in clusters responsive to anti-CD14 treatment (clusters 0, 3, 5, 7, 9). (F) Heatmap of differentially expressed genes in CCR2+ cell populations grouped by condition and row normalized genes. Only genes that were statistically significant (adjusted *P* value <0.05) between treatment and control subjects are included in (F and G). (G) Gene set score for differentially expressed genes down-regulated in anti-CD14 treatment relative to isotype control. (H) Palantir trajectory analysis pseudotime (left) and entropy (right) values in UMAP space. (I) Inflammatory branch probability across pseudotime, with cells going down a proinflammatory lineage marked in blue. (J) Temporally ordered gene expression heatmap of key pro-inflammatory genes across pseudotime along the proinflammatory lineage. Abbreviations as in Figures 1 and 2.

**Figure 5:**
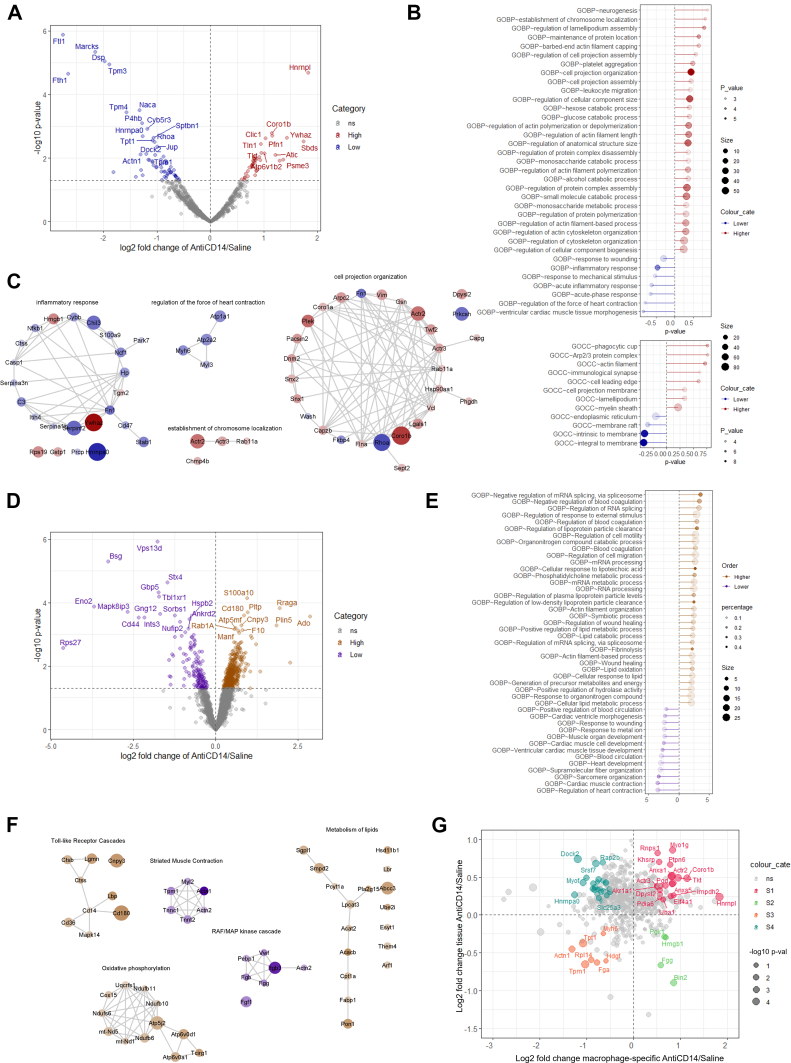
Differential Macrophage and Tissue-Level Proteomics Following CD14 Blockade at 3 Days Post-STEMI (A) Volcano plot of macrophage cell proteome of anti-CD14 treatment vs saline based on differential expression with Limma-Voom. Differential up-regulated (red) and down-regulated (blue) proteins with *P <* 0.05 are highlighted. (B) Lollipop plot of functional enrichment analysis of anti-CD14 treatment vs saline based on differential expression of macrophage cell proteome (Perseus 1D enrichment analysis; biological processes and cellular component). Statistics can be found within Supplemental Data 1 and 2. (C) Gene Ontology Component/Process/Function enrichments retrieved through STRING Enrichment in Cytoscape (*P <* 0.05). Specific proteins represented for each functional category are indicated; colors represent differential expression relative to saline, with node size representing the level of significance. (D) Volcano plot of infarcted tissue proteome of anti-CD14 treatment vs saline based on differential expression with Limma-Voom. Differential up-regulated (gold) and down-regulated (purple) proteins with *P <* 0.05 are highlighted. (E) Lollipop plot of functional enrichment analysis of anti-CD14 treatment vs saline based on differential expression (*P <* 0.05) of infarcted tissue proteome. Statistics can be found within Supplemental Data 3 and 4. (F) Gene Ontology Component/Process/Function enrichments retrieved through STRING Enrichment in Cytoscape (*P <* 0.05). Specific proteins represented for each functional category are indicated; colors represent differential expression relative to saline, with node size representing the level of significance. (G) Scatter plot of the log2 fold changes of proteins differentially expressed (*P* value <0.05; anti-CD14 treatment vs saline for macrophage or apex tissue [x-/y-axis]; differential expression with Limma-Voom). Subgroups of proteins reveal similar/opposing effect of anti-CD14 treatment on macrophage and infarcted cardiac tissue, in which similar trends in differential expression (pink/orange) or opposing trends (green/cyan) for specific proteins (*P* < 0.10) are indicated (Supplemental Data 5).

We performed experiments at 1 and 3 days post-STEMI to investigate underlying biological changes preceding the structural and functional benefits observed with anti-CD14 treatment from 7 to 28 days post-STEMI (Figures 3 to 5).

To determine whether anti-CD14 treatment impacted acute infarct size and cardiac injury, we performed Evans blue/tri-tetrazolium chloride staining of hearts and measured circulating cardiac troponin-I in a cohort of mice at 24 hours post-STEMI (Figures 3A and 3B), with statistical power of the Evans blue/tri-tetrazolium chloride staining sufficient to detect an infarct size effect of ≥15% (Supplemental Methods). We did not observe any treatment effect on acute infarct size or circulating cardiac troponin-I levels *(P =* 0.75 and 0.77, respectively) (Figure 3C).

We next assessed whether the abundance and phenotype of monocytes/macrophages within the infarcted myocardium were impacted by CD14 blockade at 3 days post-STEMI (Figure 3D). Immunohistochemistry and flow cytometry revealed that monocyte/macrophage abundance within the infarct was unaffected by anti-CD14 treatment at this timepoint (Figures 3E to 3H). Similarly, anti-CD14 treatment did not influence the numerous differences found when comparing the levels of circulating cytokines at D1 with those present at D3 (eg, IFNγ, IL-1α and β, IL-10, IL-12, TNFα, VEGF) (Supplemental Figure S6, Supplemental Table S5).

To test our hypothesis that the benefits observed with anti-CD14 treatment between 3 and 7 days post-STEMI were associated with an altered macrophage phenotype, we performed RNA sequencing of all CD11b+/CD64+/CD45+ macrophages (Figures 3G to 3H) isolated from the infarcted myocardium of each group at 3 days post-STEMI. Sequencing analysis detected numerous differentially expressed genes (DEGs) when comparing anti-CD14 antibody treatment with saline (1,585 DEGs) or isotype (1,565 DEGs) control groups (Figure 3I). Many of the 629 commonly DEGs were involved in immune responses and immunomodulation (Figure 3J). Specifically, anti-CD14 antibody treatment reduced the expression of genes involved in leukocyte trafficking (Ccl2, Ccl3, Ccl4, Ccl7) and proinflammatory macrophage responses (Tnf, Runx1, Nfkb1, Ptgs2), and increased the levels of transcripts associated with anti-inflammatory responses to injury and the protection of extracellular matrix (including C1qa/b, Cebpb, Maf, S100a6 and Timp2) (Figure 3J). Sorting nexin-20, a regulator of immune cell recruitment, and several MAPK-related genes associated with myocardial ischemia-reperfusion injury (Gps2, Map3k4, and Map4k4) were also significantly down-regulated in cardiac macrophages from anti-CD14 antibody-treated mice. Functional enrichment analysis revealed numerous processes associated with inflammation, immune cell activation/signaling, and responses to wound healing were attenuated in the infarcted LV myocardium of anti-CD14-treated animals (Figure 3K).

Following these pseudo-bulk transcriptomic analyses, subsequent single cell RNA sequencing (scRNAseq) analysis of the monocyte/macrophage transcriptome from the same set of flow-sorted cells was also performed by an independent sequencing laboratory (Figure 4). After quality control (Supplemental Figure S7), merging data and dimensional reduction, semiautomated clustering analysis revealed that there were 9 transcriptionally distinct populations of monocytes and macrophages present in the infarcted myocardium at day 3 post-STEMI (Figures 4A to 4C), with 2 small (<4% of total cell count) separate clusters of dendritic cells and neutrophils (clusters 6 and 8) (Figures 4A to 4C). The proportion of cells in cluster 0, the dominant population in the infarcted hearts in these experiments, appeared to be more abundant in the anti-CD14 treatment group, and consisted of cells with markers of mature polarized macrophages (Figure 4); however, statistical comparisons cannot be made from pooled cells (n = 5/group). The relative abundance of the Ly6c2^hi^ subpopulation (cluster 1) isolated from infarcted tissue appeared smaller in the anti-CD14 treatment group samples (21% vs 25% and 27% in saline- and isotype-treatment groups, respectively) (Figure 4C). The cell density map (Figure 4D) helps to visualize compositional changes in the isolated monocytes/macrophages, highlighting a reduced density/abundance of cluster 1 (Ly6c2^hi^) and cluster 4 (proliferating monocytes/macrophages) cells, and an increased density of cluster 5 cells (macrophages involved in heme transportation and catabolism) in the animals treated with anti-CD14. Other macrophage clusters and the dendritic cell and neutrophil populations (clusters 2, 3, 6, 7, 8, 9, 10) were similar in abundance between all treatment groups. We observed that Ly6c2^hi^ monocytes and derived macrophages isolated from infarct-tissue (cluster 1), the clusters most depleted in the anti-CD14 treatment group, highly expressed *CCR2* (Figure 4E). Conversely, the predominant cluster 0 (mature/reparative macrophage population) had low *CCR2* expression. DEG dot-plot analysis and gene set scoring of differential gene expression further indicated that anti-CD14 therapy significantly altered the genetic signature of cells within the CCR2+ populations of flow-sorted cells, including reduced expression of genes encoding proinflammatory processes in these cells (eg, Tnf, Il1b, Nfkb) (Figures 4F and 4G). To dissect the impact of anti-CD14 therapy on monocyte ontogeny and macrophage fate specification we performed trajectory analysis using Palantir and found the greatest entropy or earliest pseudotime in monocyte and certain macrophage subsets (Figure 4H). Notably, we found that the inflammatory lineage was depleted in the anti-CD14 group (Figure 4I), and expression of proinflammatory genes and transcription factors such as *Runx1* (implicated in adverse remodeling) were enriched along this trajectory (Figures 4I and 4J).

Alongside these transcriptomic effects, unbiased label-free proteomic analysis of macrophages isolated from a separate cohort of infarcted myocardium samples 3 days post-STEMI revealed down-regulated biological terms associated with inflammatory response (Hnrnpa0, Serpinf2, Chil3, Stab1, S100a9, Ctss, Serpina1b; independent of measures of plasma proinflammatory markers) (Supplemental Figure S8), as well as decreased contraction-regulatory protein networks (Map2k1, Rhoa, Atp1a1, Atp2a2), membrane-associated (transmembrane) proteins (Sptbn1, Jup), ferric iron binding (Ftl1, Fth1), and endopeptidase inhibitor activity (Serpinf2, Serpinh1, Serpina1a/b/c, Serpina3n) following anti-CD14 treatment (Figures 5A to 5C, Supplemental Figure S7). To identify corresponding transcripts from single cell analyses, we performed comparative analysis to identify 48 intersect genes down-regulated in expression (both proteome and single cell level) in macrophages following anti-CD14 therapy (Ctsl, Nfkb1). Gene expression enrichment analysis highlighted terms “leukocyte cell-cell adhesion,” “generation of precursor metabolites and energy,” “actin filament organization,” “phagocytosis,” “regulation of apoptotic signaling pathway,” and “collagen metabolic process” within this shared cluster.

Subsequently, in isolated macrophage proteome we observed an increased expression of cell-edge (shape) regulatory proteins and components, protein networks associated with chromosome localization, and protein binding to signal transduction components following anti-CD14 therapy. In addition, bulk proteome expression analysis of whole infarcted tissue samples validated transcriptionally down-regulated sarcomere/structural proteins and organization (Actn2, Mypn, Tnnt2, Tpm1, Csrp3; associated with tissue remodeling), responses to wound healing (Anxa1, Cd36, Fgf1), platelet activation, signaling (Apoh, Plg, Gna13, Pros1), signaling by receptor tyrosine kinases (Mapk14, Tns3, Csk, Hnrnpf), and iron uptake/signaling (Tcirg1, Atp6v0d1, Atp6v0a1) (Figures 5D to 5F, Supplemental Data 3 to 4, Supplemental Figure S9).

Other proteomic changes were also observed in both isolated macrophages (monosaccharide metabolic processes, and platelet aggregation) (Supplemental Figures S9K and S9L) and whole infarcted tissue (lysosomal, RNA metabolism and prostaglandin) (Supplemental Figures S9M and S9N). Comparative proteome analyses of anti-CD14 treatment in macrophage population vs bulk (global) cardiac tissue highlighted tissue and cell type-specific proteins associated with macrophage regulation (273: up-regulated, 104: down-regulated). Shared protein networks upregulated in response to anti-CD14 treatment highlighted markers of anti-inflammatory response and metabolism, including ANXA1 and ANXA5, cytoskeletal rearrangement (DOCK2, RAP2B), mitochondrial function (SLC25A3), and transcriptional regulators SRSF7 and HNRNPA0, and membrane/phospholipid binding proteins (BIN2) (Figure 5G, Supplemental Data 5). We identify cell type–specific up-regulated changes in macrophage proteome (relative to bulk tissue) linked with gluconeogenesis and cell adhesion remodeling.

These results show that cell type–specific expression profiling obtained using multi-omics can be used to link cell-intrinsic changes in expression linked with leukocyte immunoregulation, kinase signaling and regulation of inflammatory response, and other networks associated with matrix-metabolic, apoptotic signaling and regulation, and leukocyte function to highlight molecular analysis in anti-CD14 therapy following STEMI.

## Discussion

Here we report the findings of the first preclinical trial to investigate a targeted immunomodulatory strategy of CD14 blockade with an anti-CD14 neutralizing antibody given at reperfusion following STEMI. We show that initiating CD14 antibody blockade at reperfusion prevents the post-acute/chronic progression of LV systolic dysfunction, chamber dilatation, and hemodynamic decline over 28 days post-STEMI, independent of acute injury and dysfunction.

Despite increasing attention to immunomodulation strategies following myocardial infarction,^16^ previous strategies have largely failed at the bedside. Namely, nonspecific immunomodulatory treatments of patients with MI do not convincingly improve outcomes, widely attributed to their inadvertent suppression of reparative immune cell abundance and functions, and particularly those of the monocyte-derived cardiac macrophage.^16^^,^^32^, ^33^, ^34^ Treatments targeting specific modulators of the innate immune response have shown some promise in both preclinical and clinical STEMI trials.^12^^,^^35^^,^^36^ For example, IL-1 blockade has been shown to reduce the risk of heart failure in patients with MI,^14^^,^^15^ and a large clinical trial of IL-6 inhibition in patients with MI is ongoing (NCT06118281).

Importantly, the selective immunomodulatory effects observed in our reported studies of CD14 blockade were observed without monocyte/macrophage depletion (cross-validated by flow cytometry and immunohistochemistry). There were no adverse events typical of immunosuppression observed in our trials (eg, increased infections, cardiac rupture, bone fractures, excess hair growth, or slowed wound healing^37^^,^^38^) (Supplemental Table S1). This finding is in line with the emerging favorable safety profile associated with clinical trials of CD14 blockade in patients with amyotrophic lateral sclerosis (NCT04309604, NCT03487263)^29^ and COVID-19 pneumonia (NCT04391309).^31^ Phase 1b studies with Atibuclimab to treat arrhythmogenic cardiomyopathy (NCT06275893),^30^ and acute decompensated heart failure (NCT06556810) are underway.

Preclinical/translational trials in existing murine models of MI (with and without reperfusion) frequently overgeneralize their findings to suggest translatability to all subcohorts of MI patients. Widely variable (and typically modest) ischemic area/infarct sizes reported between research groups/surgeons, lack of internal validation of STEMI/non-STEMI specificity, and omission of comprehensive multimodal clinical assessments hamper clinical translatability of such trials.^34^^,^^39^, ^40^, ^41^ To minimize these limitations, for these studies we employed our center’s rigorous preclinical protocol for STEMI that reduces variation of AAR by adhering to predetermined inclusion criteria throughout all studies (obtained by clinically analogous ECG^42^ and echocardiography^43^^,^^44^). As reported herein, this protocol produces a translationally appropriate murine model of large anterior reperfused STEMI with key clinical features, including acute and progressive LV systolic dysfunction and adverse remodeling, respectively. These trials additionally leveraged multimodal translational endpoints to corroborate structural and functional observations, including serial echocardiography, CMR, invasive hemodynamics, and histopathology—internally interrogating reproducibility and informing subsequent translation.^9^^,^^45^^,^^46^ We also include the findings of subsequent focused and hypothesis-driven examinations of mechanisms analyzed by independent laboratories. Furthermore, all animals were strictly randomized and treatment blinding was maintained until all data were analyzed and reported to the trial sponsor (including adverse event and postmortem reports, see Supplemental Table S1) to maintain the highest standards for study integrity (see Data and Code Availability for access to primary data).^34^ These efforts notwithstanding, the model of large anterior STEMI with reperfusion used in these reported trials was characterized by key clinical features of patients with large anterior STEMI and therefore has limitations to its broad generalizability. Additional constraints and limitations of these studies are detailed towards the end of this discussion.

### CD14 blockade safely prevented postacute progression of LV dysfunction and remodeling, independent of acute injury

Following large anterior reperfused STEMI, control animals (both saline- and IgG2a antibody isotype-treated groups) developed moderate LV systolic dysfunction within 24 hours (partially attributable to both tissue injury and transient myocardial stunning,^47^ and the surgical approach to induction). Similar LV dysfunction was also observed at 3 days post-STEMI, which then worsened alongside progressive LV dilatation out to 28 days post-STEMI (assessed using multiple clinical modalities, including echocardiography, CMR, and intracardiac PV catheterization of the LV). Intraventricular PV hemodynamics were also impaired at both 7 and 28 days post-STEMI, including significantly reduced LV developed pressures, positive change in pressure/change in time, and stroke work observable at both timepoints (compared with sham control subjects; presented here for reference only and not included in this report’s original blinded analysis).

In these preclinical trials, CD14 blockade with a murine analogue of Atibuclimab, biG53 LALA-PG at reperfusion (with RO of >95% confirmed by separate in vivo trials, see Supplemental Figure S1), did not affect acute infarct size or early LV systolic dysfunction at 1- or 3-days post-STEMI. However, at 7-days post-STEMI, and out to 21- and 28-days post-STEMI, CD14 blockade prevented further progressive LV dysfunction and dilatation, and preserved the structural and functional state of the LV at 28-days. These benefits of CD14 blockade are well illustrated by the intraventricular PV loop characteristics of anti-CD14-treated mice, uniquely preserved out to 28-days post-STEMI compared with the progressive rightward (dilatated) and downward (hypotensive) shift observed in both control-treated groups between 7- and 28-days post-STEMI. In aggregate, these data suggest that CD14 blockade was associated with prevention of the secondary chronic progression of LV systolic dysfunction and dilatation independent of acute injury and dysfunction.

Subsequently, separate studies were carried out to explore our originally hypothesised mechanism of action for CD14 blockade, ie inhibition of secondary monocyte-macrophage activation and associated injury exacerbation following STEMI (postulated previously as a potential mechanism of therapeutic action in other models of ischemic injury^48^^,^^49^).

### Early/acute changes in the monocyte-macrophage transcriptome and proteome precede treatment benefits

Our transcriptomic and proteomic studies revealed and validated a profound down-regulation of genes and proteins associated with pro-inflammatory and remodeling processes, and preservation/upregulation of anti-inflammatory, metabolic, and injury-resolving processes with CD14 blockade.

The positive treatment effects of CD14 blockade observed from 7- to 28-days post-STEMI were transcriptionally associated with earlier selective immunomodulation of CD14^+^ cells at 3-days post-STEMI. In monocyte-macrophages isolated from the infarcted myocardium at this timepoint, CD14 blockade initiated at reperfusion had significantly downregulated >620 genes (including Tnf, Tlr2, NFkb1, Il10, Clcl1/2/10, Ccl2/3/4/7 and Ccrl2), many of which are associated with proinflammatory responses to injurious stimuli, subsequent innate/adaptive immune cell migration and adhesion, and monocyte/macrophage activation (Figure 3K). Within these isolated monocyte-macrophages, independently performed analysis of single-cell RNA sequencing revealed that CD14 blockade also resulted in a selective decrease in abundance of pro-inflammatory *Ly6c2*^*hi*^ *CCR2*^*+*^ monocytes and macrophages (Cluster 1) in the infarcted myocardium, and an increase in *CCR2*^*-*^ cells expressing markers of macrophage maturation, repair and polarization (Cluster 0), similar to those described previously as “late/transient-macrophage-2”^50^ or “non-inflammatory” “transient/fully-differentiated” macrophages.^51^ CCR2^-^ macrophages have been associated with reparative or maintenance processes, while CCR2^+^ macrophages are involved in adverse remodeling, fibrogenesis, arrhythmia and contractile dysfunction.^52^ Ly6c2^hi^ (Cluster 1) monocytes and macrophages, the population that we found to be decreased in abundance after anti-CD14 treatment, are the closest murine equivalent of the highly pro-inflammatory and migratory immune CD14^++^ CD16^-^ classical macrophages (that are also CCR2^+^) associated with injury and chronic disease in humans.^52^^,^^53^

Proteomic analysis of macrophages isolated from the infarcted myocardium 3-days post-STEMI largely validated our transcriptomic findings (Figures 3 and 5, Supplemental Figures S5 and S7). The isolated macrophage proteome revealed a similarly downregulated inflammatory response with anti-CD14 treatment as well as decreased contraction-regulatory and transmembrane proteins, and ferric iron binding associated with the classical pro-inflammatory macrophage phenotype.^54^ Increased expression of proteins associated with binding to signal transduction components with anti-CD14 treatment similarly validated our transcriptomic findings.

Proteomic sequencing of whole infarcted tissue biopsies revealed both similar and unique expression differentials to those observed in our transcriptomic and proteomic analyses of the isolated macrophages. These changes included down-regulated proteins associated with tissue remodeling, responses to wound healing, and MAPK signaling. Interestingly, the proteome of the infarcted myocardium was revealed to have decreased expression of cardiac muscle contraction with anti-CD14 treatment, suggestive of reduced compensatory hyperkinesis in viable cardiomyocytes within these tissues. Observed differentials in proteins related to muscular function accompanied an increase in whole-tissue markers of lipid metabolism, potentially indicating myocardial salvage postischemia/hypoxia with anti-CD14 treatment. Our comparative analyses of the macrophage-specific vs global proteomes suggest that these changes in metabolism were predominantly up-regulated in nonmacrophage cells. This 2-factor analysis also highlighted various markers of an up-regulated anti-inflammatory response, cytoskeletal rearrangement, transcriptional regulators, and membrane/phospholipid binding proteins common to both isolated macrophages and surrounding cells/tissue within the infarct. Of note, the temporal resolution afforded by this trial’s substudies with endpoints 1, 3, 7, and 28 days post-STEMI enabled associations between early immunological and later functional effects of CD14 blockade. Chiefly, the temporal relationships between these data suggest that these treatment benefits observed at 7 to 28 days post-STEMI were wholly independent of acute injury and dysfunction observed at 1 and 3 days post-STEMI, ie, only postacute progression of LV systolic dysfunction and dilatation secondary to the acute infarct was inhibited by CD14 blockade. This finding supports our original hypothesis that CD14 blockade initiated at reperfusion modulates the known delayed monocyte/macrophage response to injury, which develops between days 2 and 4 post-STEMI.^19^ Despite a similar abundance of CD68+ monocyte-macrophage infiltrates between groups observed at 3 days post-STEMI, anti-CD14 treatment uniquely reduced the abundance of these cells 7 days post-STEMI, prior to complete resolution below detectable levels at 28 days post-STEMI (Figures 3E and F). This finding suggests that the observed modulation of the early monocyte/macrophage response at 3 days post-STEMI ultimately preceded an expedited resolution of this immune cell involvement over subsequent weeks. This may be partly explained by the down-regulation of CCL2 (ligand for CCR2) and other proinflammatory/chemotactic ligands observed with CD14 blockade (Figure 3J). Dedicated flow cytometry studies are needed to interrogate whether this is associated with decreased recruitment of circulating monocytes or expedited efflux of tissue resident macrophages post-STEMI (Figures 4B and 4C). In any case, we observed decreased expression of genes associated with chemotactic signals in these monocytes/macrophages, which preceded the observed reduction in total cardiac macrophage abundance at day 7 post-STEMI (Figures 3E and 3F). Alternatively, the arrival and dominance of cluster 0 cells (reparative, fully mature macrophages) within the infarcted LV by 3 days post-STEMI may have been accelerated by anti-CD14 treatment,^50^^,^^51^ but further experiments are required to explore this possibility.

We next performed integrative analysis of isolated macrophages at the transcriptome and proteome level (down-regulated by anti-CD14 treatment) revealing enrichment in gene ontology terms related to myeloid leukocyte-mediated immunity (migration, adhesion, proliferation, and response) (Supplemental Figure S11). This multi-ome analysis combined with multimodal physiological phenotyping support our principal hypothesis that anti-CD14 blockade selectively modulates the macrophage cell landscape in the absence of macrophage depletion to preserve LV function following STEMI. Whole infarcted tissue proteome analysis, although revealing tissue-level down-regulation of sarcomeric/structural protein reorganization and responses to wound healing, was unable to provide the resolution to identify these macrophage-centric changes at the cellular level. From these integrative analyses, we infer a central role of macrophage identity and function directing the progressive immune-mediated remodeling of the infarcted myocardium.

### Study limitations

#### Control groups

The saline control group included in these preclinical studies serves well as an analogue to a double-blind “placebo” in clinical studies of STEMI interventions. By comparison, the isotype control used in these studies is not a perfect isotype of the anti-CD14 antibody being investigated, and this may limit interpretation of comparisons between anti-CD14 and the isotype control. Although both the anti-CD14 antibody used in this study and its isotype control mAb are mouse IgG2a antibodies, this murine anti-CD14 antibody contains a LALA-PG Fc domain mutation that was not able to be engineered into the IgG2a control antibody at the time of these experiments. The LALA-PG mutation reduces Fcγ receptor binding thus preventing antibody-dependent cellular cytotoxicity, antibody-dependent cellular phagocytosis, blocks binding to complement component C1q, thereby preventing complement-dependent cytotoxicity and preserves binding to the neonatal Fc receptor (FcRn), which is responsible for recycling IgG and maintains its long serum half-life. In contrast, the isotype antibody used in these studies was a commercially purchased IgG2a antibody which has no LALA-PG mutation in the Fc region. Subsequent experiments in different cardiac indications used an IgG2a LALA-PG isotype control that we have developed following the work contained in this paper. We posit that Fc interactions are the reasons for the observed differences between saline and isotype control data, and may explain the additional variance/skew observed in the data of the isotype control group for some parameters.

#### Outstanding mechanistic questions for further investigation

There are some limitations associated with these preregistered preclinical trials designed to chiefly investigate clinical/translatable outcomes. Our reported substudies interrogating the underlying potential mechanism/s of action were designed to test the specific hypothesis that anti-CD14 antibody blockade selectively modulates the secondary proinflammatory monocyte/macrophage response to ischemic cardiac injury. We comprehensively tested this hypothesis using preregistered protocols involving flow cytometry, immunohistochemistry, transcriptomic (including single-cell RNA sequencing), and quantitative proteomic techniques, using tissues acquired from independent cohorts of animals post-STEMI that were separate from those used for functional trials. Despite the advantages of this hypothesis-driven approach, additional questions raised by these findings were not investigated and absolutely warrant further investigation.

For example, further experimentation is needed to increase the temporal resolution of monocyte/macrophage trafficking dynamic studies following CD14 blockade. Further, to comprehensively describe cellular trafficking dynamics, further interrogation of the source of relative monocyte/macrophage clusters observed in the infarcted myocardium is needed (be they tissue resident or recruited cells, or both), eg, subsequent expanded flow cytometry studies dedicated to quantifying the absolute abundances of salient monocyte/macrophage subpopulations of interest, informed by our scRNAseq data. Some of these studies are perhaps best performed using large animal models where serial sampling can feasibly and safely be performed through endocardial biopsy and blood draw. Additionally, although the observed modulation of the predominantly abundant monocyte and macrophage populations is most likely to be the primary driver of the delayed structural and functional benefits of anti-CD14 treatment, the potential involvement (crosstalk and immunomodulatory contribution) of similarly modulated acute infiltrates of CD14+ dendritic cells and neutrophils may precede these changes and warrants further investigation.^55^^,^^56^ Another question is to what degree the anti-CD14 antibody penetrates and persists within the cardiac tissue, and whether the effects seen are through a systemic or direct cardiac effect, or both. As yet, we have not been able to assess CD14 antibody receptor occupancy in cardiac tissue macrophages, because their binding sites are affected by the isolation procedure. We posit that it is likely the recruited monocyte-derived macrophages (rather than tissue macrophages) are the cell population that is selectively affected by CD14 blockade, and lineage tracing techniques would be informative in future trials of anti-CD14 in STEMI. It should be noted that low levels of CD14 may be expressed by other cardiac cell types such as cardiomyocytes^57^ or activated fibroblasts (CD14 expression not observed in vivo),^58^ but it is possible that these fibroblasts may also be derived from macrophages.^59^ These new questions were not the focus of these trials but are key areas of interest in this field to be answered by dedicated cell trafficking, proliferation, and signaling studies in STEMI.

## Conclusions

Principally, these data provide the first evidence of safety and efficacy of a clinically practicable targeted immunomodulatory strategy of CD14 blockade in a translationally appropriate preclinical model of STEMI with key clinical features. The findings of this blinded and randomized preclinical trial demonstrate that anti-CD14 antibody treatment initiated at reperfusion inhibited the chronic immunological progression of LV systolic dysfunction, dilatation, and haemodynamic decompensation post-STEMI, independent of acute injury and dysfunction. Our findings additionally provide new and novel transcriptomic and proteomic insights into the upstream regulatory role of CD14 in the immunological progression of ischemic heart failure, and inform future stages of both basic and translational research into CD14 blockade.

Perspectives **COMPETENCY IN MEDICAL KNOWLEDGE:** STEMI is a severe and life-threatening presentation of MI. Inhibiting the proinflammatory activities of monocytes and monocyte-derived macrophages following MI has been proposed as a key area for modern intervention, but immunosuppression or monocyte depletion is not a viable therapeutic strategy. Our present studies explored the use of a CD14-neutralizing antibody therapy (a murine analogue of Atibuclimab, recently found to be safe in clinical trials). In a translational murine model of large anterior reperfused STEMI, we found that CD14 blockade initiated at reperfusion prevented chronic progression of cardiac dysfunction and dilatation, independent of acute injury and dysfunction. Preceding these observed treatment benefits, CD14 blockade selectively down-regulated proinflammatory processes in cardiac macrophages without suppression of their infiltration or reparative processes. Informing future clinical trials, these preclinical findings support CD14 blockade, initiated at reperfusion post-STEMI, as a practicable targeted immunomodulatory strategy for the prevention of secondary immunological exacerbation of acute myocardial injury postinfarction.

**TRANSLATIONAL OUTLOOK:** These preclinical trials evaluated anti-CD14 antibody treatment initiated at reperfusion in a translational model of STEMI with key clinical features using multiple clinical modalities. CD14 blockade prevented postacute progression of left ventricular systolic dysfunction, remodeling and hemodynamic decompensation associated with secondary immunological injury, independent of acute infarct size and dysfunction. These findings provide the first evidence of a practicable and translatable targeted immunomodulatory strategy of CD14 blockade initiated at reperfusion to prevent chronic immunological progression toward ischemic heart failure, and inform the design of subsequent clinical trials.

### Data Availability Statement

All supporting data are available within the main paper and Supplemental Material or available from data repositories. Proteomic data (RAW and processed/search files) for global (apex) and tissue macrophages are available from the Proteome Xchange Consortium via the PRIDE partner repository with the data set identifier PXD041192. Proteome intensities were processed via VSN normalization method (Limma package) in R. For proteomics analyses, the Human Protein Atlas (https://www.proteinatlas.org/human proteome/tissue) and functional enrichment annotations using g:Profiler (https://biit.cs.ut.ee/gprofiler/) were used. Further pathway enrichment map analysis was performed using Cytoscape (version 3.7.1), Reactome, and DAVID functional annotation software. Specifically for macrophage proteome, t-statistic of DE analysis was subjected to 1D enrichment analysis in Perseus software for functional enrichment of Gene Ontology Biological Processes (GOBP), Cellular components (GOCC) and Molecular Functions (GOMF). For cardiac apex proteome, differentially regulated proteins (*P <* 0.05) were used for functional enrichment analysis in Cytoscape StringApp focusing on gene ontology (GOBP, GOCC, GOMF) and pathways (KEGG, Reactome, Wikipathway). Data visualizations were completed using either Cytoscape or R (ggplot2 package).

## Funding Support and Author Disclosures

This trial was sponsored by Implicit Bioscience Ltd, which provided contract research funds and materials (anti-CD14 antibody) with untethered scientific freedom. Support was also provided by the National Health and Medical Research Council and National Heart Foundation (postgraduate scholarships to Dr Bloom), National Health and Medical Research Council (fellowship to Drs Wright and Kaye), Shine On Foundation (Senior Research Fellowship to Dr Donner), Victorian Government Operational Infrastructure Support Program, and the Rebecca L. Cooper Medical Research Foundation. Dr Abbate has received research support from Implicit Bioscience Ltd. Drs Appleby, Crowe, Redlich, and Ziegelaar are employees of the trial sponsor (Implicit Bioscience Ltd), which maintains a commercial interest in the anti-CD14 monoclonal antibody, Atibuclimab (IC14). Drs Redlich and Ziegelaar are named inventors on patent WO2022051814A1 relating to the use of CD14 antagonist antibodies for treating myocardial infarction. All primary trial data was recorded, analyzed, and reported while blinded by the Baker Heart and Diabetes Institute and the study director (Dr Donner), independently of Implicit Bioscience Ltd. All other authors have reported that they have no relationships relevant to the contents of this paper to disclose.
