# Supplementary material for: CD14 Blockade Modulates Macrophage-Mediated Immunological Injury in a Translational Model of Reperfused ST-Segment Elevation Myocardial Infarction
Source: JACC Basic Transl Sci. 2025 Oct 23;10(11):101393. doi: 10.1016/j.jacbts.2025.101393 (PMC12790148; doi:10.1016/j.jacbts.2025.101393)
Supplement: Supplementary Material [file mmc1.docx]

## SUPPLEMENTARY MATERIALS CD14 Blockade Modulates Macrophage-Mediated Immunological Injury in a Translational Model of Reperfused ST-Segment Elevation Myocardial Infarction.

Aascha A D’Elia (nee Brown), PhD^1^; Helen Kiriazis, PhD^1,15^; Jason Bloom, MD^2,9^; Jonathan Noonan, PhD^3,10,15^; Ian Hsu, PhD^4^; Gabriella E Farrugia^4^, PhD; Haoyun Fang, PhD^5^; Stephanie Jansen, BVetSc^16^; Natalia Carvajal, PhD^12^; Crisdion Krstevski, PhD ^4^; Waled A Shihata, PhD^2^; Yow Keat Tham, PhD^6,10,15,18^; Angela Vais, PhD ^11^; Camilla Cohen, PhD ^11^; Adam C Parslow, PhD^8,15,18^; Chad Johnson, PhD^17^; Anita C Thomas, PhD^1,9^; Malathi I Dona, PhD^4^; Kyah Grigolon, BVetSc^1^; Scott JY Loh, BSc^1^; Guy Krippner, PhD^1,15^; David K Wright, PhD^14^; Bing H Wang, PhD^7,10^; Antonio Abbate, MD^,^, PhD^19^; Junedh Amrute, PhD^20^; Kory Lavine, MD, PhD^20^; Mark W Appleby, PhD^21^; David Crowe, PhD^21^; Garry Redlich, DUniv ^21^; Brian W Ziegelaar, PhD^21^; Julie R McMullen, PhD^6,10,13,15^; David W Greening, PhD^5,10,15,18^; Alexander R Pinto, PhD^4,10,15,18^; David M Kaye, MD, PhD^2,9,10^; Daniel G Donner, PhD^1,9,10,15^

^1^Translational Cardiology Centre, ^2^Heart Failure Laboratory, ^3^Atherothrombosis & Vascular Laboratory, ^4^Cardiac Cellular Systems Laboratory, ^5^Molecular Proteomics Laboratory, ^6^Cardiac Hypertrophy Laboratory, and ^7^Biomarker Discovery Laboratory, ^8^Baker Institute Microscopy Platform, Baker Heart and Diabetes Institute, Melbourne, Australia, ^9^Department of Cardiology, Alfred Hospital, Melbourne Australia, ^10^School of Translational Medicine, ^11^Monash Histology, ^12^Monash Sequencing, ^13^Department of Physiology, and ^14^Department of Neuroscience, Monash University, Melbourne, Australia,^15^Baker Department of Cardiometabolic Health, University of Melbourne, Melbourne, Australia,^16^Alfred Medical Research and Education Precinct Animal Services, Melbourne, Australia, ^17^Bioimaging Platform, La Trobe University, Melbourne, Australia, ^18^Baker Department of Cardiovascular Research, Translation and Implementation, La Trobe University, Melbourne, VIC, Australia, ^19^Robert M. Berne Cardiovascular Research Center, University of Virginia, Charlottesville, VA, USA, ^20^Departments of Medicine; Pathology & Immunology; and Developmental Biology, Washington University School of Medicine, St Louis, MO, USA, ^21^Implicit Bioscience Ltd., Brisbane, Australia and Seattle, WA, USA.

## Data and Code Availability

All supporting data are available from data repositories. All primary data are available upon request from the corresponding author or trial sponsor.

Supplementary Data 1 – Cardiac macrophage-specific proteome (analyzed using Limma package)

Supplementary Data 2 - Enrichment analysis of macrophage-specific anti-CD14/saline

Supplementary Data 3 - Cardiac tissue proteome (analysed using limma package)

Supplementary Data 4 - Enrichment analysis of cardiac tissue anti-CD14/saline

Supplementary Data 5 - Two-way analysis of cardiac tissue and macrophage-specific proteome

# Supplementary METHODS

## Development of a murine anti-CD14 antibody for preclinical trials

To achieve CD14 blockade in mice, we developed and utilized a non-depleting anti-mouse CD14 IgG2a monoclonal antibody (mAb) with 3 Fc region silencing mutations to cripple ADCC, CDC & ADCP function (biG53 LALA-PG; Implicit Bioscience Ltd, Seattle, WA, US)[1]. We expressed biG53 LALA-PG mAb in CHO cells and affinity-purified to >95% purity (Fusion Antibodies, Belfast, UK). Inflammatory cytokine suppression with biG53 LALA-PG was evaluated by lipopolysaccharide (LPS) cytokine assays (360biolabs, Melbourne, AU). Receptor occupancy studies were performed (Hooke Laboratories, Lawrence, US) and the pharmacokinetic properties of biG53 LALA-PG mAb were determined following a single 5 mg/kg IP dose (at day 0) in C57BL6 mice. Serum biG53 LALA-PG levels were determined by capture ELISA (Hooke Laboratories) with data analysis using Phoenix WinNonlin software (Certara, Princeton, US).

## Murine STEMI model of progressive LV systolic dysfunction and remodeling

***In Vivo Pilot Study to Establish Dose Range:*** A pilot study was used to confirm an effective dose range (echocardiographic ejection fraction [% EF] 7 days post-STEMI as the primary endpoint, see **Supplementary Figure S2**), and for required sample size calculations for subsequent trials (SigmaPlot V10.0, Systat Software Inc., DE), expected [observed] difference in means: 5% EF, expected [observed] SD of residuals: 4% EF, number of groups: 3, desired power: 0.8 and alpha: 0.05. Computed sample size for one-way ANOVA = 14 per group).

A dose of 5 mg/kg IV anti-CD14 antibody (in 150 µl phosphate buffered saline) was used across all trials, except for the 28-day post-STEMI endpoint trial where 5 mg/kg weekly anti-CD14 dosages (given each week for 4 weeks) were given to maintain adequate receptor occupancy (see **Supplementary Figure S1**) throughout the extended trial term. The same doses and volumes of IgG2a isotype in/and phosphate buffered saline vehicle were used across all trials as negative controls.

A total of 244 adult C57BL6 male mice (10±1 weeks of age; AMREP AS, Melbourne, AU) with STEMI were included in these studies. Multi-level randomization and blinding protocols were implemented across all studies. Briefly, mice underwent STEMI surgery and were randomly allocated to groups receiving independently randomized, prepared and blinded treatments (i.e. 150 µl saline, isotype or anti-CD14 coded as A, B or C uniquely for each separate sub-study). Accordingly, all procedures were performed, all samples/images/traces were collected, all data were statistically analysed, and each trial (Pilot, D7, D28, D1 and D3; in order of completion) was individually reported to the trial sponsor while coded treatment blinding was maintained. An additional 10 age-matched sham-operated control animals (complete surgical protocol minus vessel occlusion) have been included in figures below where appropriate (n=5 in 7-day, and n=5 in 28-day post-STEMI trial) to provide reference values for readers only. These controls were analysed post-hoc after unblinding and omitted from original statistical analyses, presented herein. For cohort sizes, see **Supplementary Table S1.** All procedures were performed at approximately the same time of day across all cohorts to limit diurnal variation in the inflammatory response.

*Model of Anterior ST-****segment Elevation Myocardial Infarction (STEMI) with Reperfusion:*** To investigate the efficacy of CD14 blockade in ischemic heart failure, we refined an existing preclinical model of reperfused MI[2, 3] with hallmarks of clinical STEMI, including classical ST-elevation,[4] and progressive LV systolic dysfunction, dilatation and haemodynamic decompensation.[5]

Briefly, mice underwent a surgical procedure to have the left anterior descending coronary artery reversibly ligated for 1 h to induce ischemia followed by reperfusion, confirmed by classical ST-elevation by EKG at each surgery (**Figure 1A, text**). Additionally, quantitative spatial assessment of stunned LV myocardium by echocardiographic endocardial displacement mapping (a non-invasive surrogate of planimetric area-at-risk; AAR, **Supplementary Figure S3**) was performed 24 h post-surgery and confirmed the predetermined inclusion criteria for each animal (i.e. AAR of 35-55 %, inclusive; **Supplementary Figure S3**).

In detail: Mice were anesthetized with a single IP injection Ketamine/Xylazine/Atropine cocktail (K: 80-100 mg/kg, X: 16-20 mg/kg, A: 1-1.2 mg/kg (Troy Laboratories Pty Ltd, Glendinning, AU)). Once anesthetized, each mouse was intubated with a tracheal cannula, shaved, prepared for surgery with chlorhexidine (5%; Perrigo Australia, Bolcatta, AU). The mice were placed onto a heated surgical table and ventilated using a Minivent mouse ventilator (150 strokes/min, 225 – 250 ul stroke volume, Hugo Sacks Elektronik, **March-Hugstetten**, DE). A single intradermal injection of local anesthetic (bupivacaine, 2 mg/kg; Pfizer Inc, New York, US) was provided at the surgical site. The chest was opened via left thoracotomy (3-4 mm incision) and retracted (6-8 mm). The left anterior descending coronary artery was visualized by surgical microscope (Opmi, Pico Zeiss; Carl Zeiss Meditec AG, Jena, DE), ligated (7-0 silk suture, Ethicon LLC, San Lorenzo, US), and tied through exteriorized releasing loops. The chest and skin were then closed (6-0 prolene; Ethicon LLC, San Lorenzo, US), and xylazine reversed (single injection of atipamezole: 0.2 mg/kg, SC; Zoetis Australia Pty Ltd, Rhodes, AU). Mice were then provided diuretic (single injection of furosemide: 3-5 mg/kg, SC; Troy Laboratories Pty Ltd, Glendinning, AU), transferred to an advanced care suite to continue constant ventilation, core temperature monitoring (rectal probe) and management, and EKG recording to confirm ST-elevation during the ischemic period. After 60 minutes of ischemia, exteriorized loops were pulled to release the ligation, immediately following administration of a single 150 ul IV bolus of phosphate-buffered saline (Sigma-Aldrich, Stenheim, DE), [saline vehicle, 0.9 % w/v], 5 mg/kg IgG2a [isotype control in vehicle], or 5 mg/kg anti-CD14 [in vehicle]. A second dose of antibody or control treatments were given 24 h later in all cohorts except for the 28-day studies (where single, weekly injections were administered either IV [at reperfusion] or IP [at subsequent doses]).

Approximately 20-24 surgeries were performed per cohort. To maximize study rigor[6], each cohort was randomized and independently blinded treatments were equally distributed within each cohort to account for risk of batch effect. During randomization of each study, sham-operated animals were included for surveillance of temporal reproducibility of the model between cohorts (assessed by 24 h echocardiographic assessment of AAR, described below. (Data presented in **Supplementary Figure S3**). Sham animals had all steps of the STEMI surgery performed except for ligation of the coronary artery and were administered 150 ul IV bolus of 0.9 % phosphate buffered saline.

***Predefined inclusion/exclusion criteria applied to each trial (including pilot study):*** All reported trial protocols, experimental methods and outcome measures were pre-registered with the trial sponsor (Implicit Bioscience Ltd, Australia) prior to commencing experiments. Along with treatment randomization and blinding, predefined inclusion and exclusion criteria were applied throughout all studies presented in this report.

Whole animal datasets were included based on the following two criterion:

- *Qualitative:* Presence of ST-elevation observed (immediately post-MI surgery, **Figure 1, text**), and
- *Quantitative:* Relative Negative Wall Displacement between 35 – 55% (24 h echocardiography, **Supplementary Figure S3**).

Factors associated with endpoint technical insufficiency were pre-defined as the only grounds for endpoint data exclusion:

- *Endpoint data:* Technically insufficient imaging/recording for analysis e.g. unsuccessful catheter insertion, computer failure/crash, unsuccessful histology sectioning.

Mice that either did not survive or were euthanized within the first hour post-surgery (i.e. before treatment) were excluded from analysis. Note: All deaths in these trials occurred prior to treatment due to acute surgical complications i.e. no animals (0) died after receiving saline/isotype/anti-CD14 prior to their planned endpoint.

## *In vivo* procedures

***Electrocardiogram (EKG; D0 <15 mins post-STEMI surgery):*** 3-lead EKG needle probes were placed under the skin to record up to 5 mins of EKG tracing (AD Instruments, Bella Vista, AU) to confirm ST elevation immediately post-STEMI surgery.

***Echocardiography (D1, D3, D7 (incl. pilot) and D28 post-STEMI):*** Mice were anaesthetized with isoflurane (4.0% induction, 1.6-1.8% maintenance; Pharmachem, Eagle Farm, AU) and comprehensive single-plane echocardiography studies of left ventricular (LV) systolic function were performed using the Vevo 2100 system (VisualSonics, Fujifilm, CA) by a senior imaging specialist.

D1 echocardiography was analyzed to confirm ischemic area homogeneity, using our validated endocardial displacement mapping technique, an emerging gold standard for the screening of MI model homogeneity, and surrogate for post-mortem planimetric AAR determination at our centre. All analyses of ultra-high-frequency parasternal long-axis loops (EKG-gated Kilohertz Visualization [EKV] for D1 wall displacement mapping) were critically performed offline by two independent observers[7], using the manufacturer software (VevoLab V5.0, VisualSonics, Fujifilm, CA). During the wall displacement mapping method for AAR measurement the endocardial circumference was traced to track and map tissue displacement in a single cardiac cycle (**Supplementary Figure S3A)**. The area of hypokinesis (i.e. infarction area) was measured and is presented as a proportion of the entire. Inclusion criteria (assessed non-invasively) were evidence of ST elevation immediately post occlusion of the coronary artery and the presence of an AAR between 35-55% at 24 h post-surgery. Any animals that failed to meet these criteria (i.e. had either a small or very large infarct) were excluded.

***Cardiac catheterization and hemodynamic assessment (D7 and D28 post-STEMI):*** Mice were anesthetized with isoflurane (4.0% induction, 1.6-1.8% maintenance) and an intracardiac catheter (SPR-839 Millar, Houston, US) was passed via the right carotid artery into the ascending aorta to measure arterial pressures, before being advanced into the left ventricle to measure left ventricular pressures and conductance. End-systolic and end-diastolic PV relationships were observed by compressing the abdominal aorta through the sub-hepatic space. Parallel conductance was corrected for using hypertonic saline infusion into the right jugular vein (5-10 µl) prior to cardiac puncture. Blood was used to construct a conductance standard curve in calibration cuvette wells of known volumes. Comprehensive hemodynamic analysis was performed offline and validated (**Supplementary Figure S8)**.

***Cardiac magnetic resonance (CMR) imaging (D21 post-STEMI):*** For superior spatial resolution assessments of LV structure and function,[8-10] CMR was performed in a cohort of animals at 21 days post-STEMI in the 28-day endpoint cohort. Mice were anaesthetized with isoflurane (4.0% induction, 1.6-2.0% maintenance) and CMR imaging was performed using a 9.4 T Magnetic Resonance Imaging system. Sequences were developed and performed using Paravision 360 V3.3 (Bruker, Karlsruhe, DE). The temperature of the mice was maintained by a circulating warm water blanket, and leads and probes applied (3-lead surface EKG, rectal probe, respiration pad). Following localizer and planning scans, brightblood sequences were used to establish 2-chamber, 4-chamber, and then short-axis views of the heart. A 5-section stack of short-axis blackblood cine images was acquired for each mouse using intragate to achieve 10 timeframes for each section. All CMR images were analyzed using Segment V3.2, R8757 (Medviso, Sweden). An example of CMR imaging analysis can be seen in **Supplementary Figure S9.**

***Blood sampling (D1 and D3 post-STEMI):*** A closed-chest heparinized cardiac puncture was performed (via the right ventricle) under anesthetic in order to collect whole blood for plasma spin-down and storage at -80^o^C before further examination.

## Post-mortem assessments

***In situ staining for area-at-risk/infarct size measurement (D1 post-STEMI):*** Following cardiac puncture, the chest was opened and ascending aorta cannulated for infusion of the coronary arteries with saline followed by retying of the ligation and infusion of 3% Evans blue dye (Sigma-Aldrich, Stenheim, DE) before heart excision. LVs were dissected and snap frozen. Later the LVs were cut into 6-8 sections of ~1 mm thickness and incubated in Evans blue/1% tri-tetrazolium chloride (EB/TTC; Sigma-Aldrich, Stenheim, DE) for 1 h at 37^o^C. Heart sections were then imaged on a flatbed scanner (both sides averaged) and analyzed by semi-volumetric planimetry (Fiji, ImageJ V1.53; NIH, US) to measure areas of total tissue (red + white + blue), ischemic tissue/AAR (red + white/beige) and infarcted tissue (white/beige). At least 15 hearts per group were included in these infarct assessment studies to detect an effect size (infarct % AAR) of ≥ 15 % (α 0.05, β 0.8).

***Autopsy and tissue collection (D1, D3, D7 and D28 post-STEMI):*** Animals were killed using an excess of anesthetic, and/or exsanguination, and/or removal of organs under anesthesia. Comprehensive autopsies were performed for all mice, including weighing of lungs, kidney, liver and spleen, measuring of tibia lengths and recording of post-mortem observations. Blinded visual assessments for presence of atrial thrombus, lung congestion, evidence of infection, and cardiac (ventricular) rupture were recorded.

***Heart collection for dissection (D3, D7 and D28 post-STEMI):*** Following cardiac puncture, a heart dissection was performed to separate, clean and weigh individual chambers. A mid-ventricular transverse ring of the left ventricle was committed to histology (detailed below), and apical/infarcted ventricle stored at -80^o^C for future experiments.

***Circulating Growth Factor measurements (D1 and D3 post-STEMI):*** Frozen plasma samples were analyzed using quantitative cytokine array 32-plex Discovery Assay® (Eve Technologies, Calgary, CA).

***Left ventricle histology (D3, D7 and D28 post-STEMI):*** Complex sectioning was performed to produce replicate slides of left (mid-) ventricular sections. Tissues were processed and embedded in paraffin. Four um thick sections were cut, stained, imaged, and analyzed by blinded personnel. Histological staining using Masson’s trichrome and picrosirius red was performed on single left ventricular section per heart each, along with fluorescence immunohistochemistry using antibodies recognizing CD68 (monocytes/macrophages) or troponin T (cardiomyocytes), with the nuclei identified using DAPI. Immunofluorescent imaging was performed at 20x magnification with an VS120 Slide-scanning Microscope (Evident (Olympus Scientific Solutions), Tokyo, Japan). All histology was analyzed for positive staining area (brightfield imaging), or positive cell count and proportion (%, immunofluorescent imaging) using Fiji (ImageJ V1.53; NIH, US). Suppliers of (immuno)histology reagents can be found in **Supplementary Table S2**.

***Flow cytometry and cell sorting (D3 post-STEMI):*** LV apexes from each heart were dissected and placed in cold 1X HBSS (14025092, Gibco; ThermoFisher Scientific, Grand Island, US,). Using scissors, tissue was minced into ~1 mm sized pieces and placed in 5 ml Eppendorf tubes containing 1 ml cold 2 mg/ml collagenase IV (CLS4, Worthington Biochemical Corporation, Lakewood, US) and 1 mg/ml Dispase II (04942078001, F. Hoffmann-La Roche AG, Basel, CH) in phosphate-buffered saline supplemented with 0.9 mM CaCl_2_ (Sigma-Aldrich, Stenheim, DE). Tissue was digested in a 37°C water bath for 45 minutes during which trituration was performed using a 1 ml pipette at 15-minute intervals. The cell suspension was filtered through a 75 µm mesh, atop 50 mL conical tubes placed on ice, into 45 mL of phosphate-buffered saline with 0.9 mM CaCl_2_. Debris clearance was performed at 200×g, 4°C for 15 minutes at maximum acceleration but with the brake off. The pellet was left in ~5 ml of volume after aspiration and an additional 1 ml of 2% FCS (heat inactivated and filtered; ThermoFisher Scientific, Grand Island, US) in 1X HBSS was added for resuspension. Cells were centrifuged at 400×g, 4°C for 4 minutes with maximum acceleration and braking, and resuspended in 2% FCS in HBSS for antibody staining.

Cells were identified using antibodies and viability dye (DAPI). Prior to flow cytometry and sorting, cells were strained through 35 µm filter tops into FACS tubes (Corning, 352235, Sigma-Aldrich, Stenheim, DE). Flow cytometry was performed on a BD LSR FortessaTM X-20 Special Order (BD Biosciences, San Jose, US) at the Baker Heart and Diabetes Institute and analyzed using FlowJo (v10.8.0; BD Biosciences, Ashland, US). Viable leukocytes were gated using DAPI exclusion (DAPI-) and CD45+ staining. Leukocyte subtypes were identified using antibodies recognizing CD11b, CD14, CD64, Ly6G, MHCII and CD206 (**Supplementary Figure S10** and **Supplementary Tables S3 and S4**).

***Single cell (macrophage-specific) RNA sequencing (D3 post-STEMI):*** Sorting of macrophages for scRNAseq was performed on a BD FacsARIA Fusion Cell Sorter (BD Life Sciences, San Jose, US) at the Alfred Research Alliance Flowcore. For each treatment group, ~10,000 cells were loaded into each lane for single cell partitioning in the 10x Genomics Chromium Controller (10x Genomics, Pleasanton, US). Gene expression libraries were prepared using Chromium Next GEM Single Cell 3’ v 3.1 Gene Expression kit (PN-1000268; 10x Genomics, Pleasanton, US), microfluidic chip (PN-1000127, 10x Genomics, Pleasanton, US) and indices (PN-1000215, 10x Genomics, Pleasanton, US) following manufacturer guidelines. Libraries were sequenced on the NovaSeq6000 (Illumina, San Diego, US) at the Alfred Research Alliance Sequencing Platform at a read depth of ~50,000 reads per cell. ~6,000 cells and ~3,500 genes/cell were recovered from each group.

CellRanger output was used to create Seurat objects (v4) in R and data from three libraries was merged into a single Seurat object. Quality control was performed and cells with 500 < nFeature_RNA < 7000, percent.mt < 10 (**Supplementary Figure S5)**, and nCount_RNA < 50000 were kept for downstream analysis. Merged data was then normalized using SCTransform regressing out percent.mt and nCount_RNA, then a principal component analysis (n = 50), uniform manifold approximation (UMAP) embedding, and subsequent clustering was performed. Differential expression analysis was used to identify non-myeloid contaminating populations which were then removed. After removing these contaminating clusters, normalization, PCA, UMAP, and clustering was re-computed. DE analysis was performed at various cluster resolutions and a heatmap of cell state marker genes constructed. For each myeloid cell state, z-scores for the top 5 marker genes were calculated and overlaid on the UMAP embedding to assess cell state separation.

Comparing across conditions: A cell composition plot of cell states was computed to identify changes in cluster composition across 3 experimental conditions. To observe phenotype shifts with anti-CD14 treatment, density plots were computed and overlaid in UMAP space using scanpy. To dissect changes in the transcriptional signatures after anti-CD14 treatment relative to isotype control, a differential expression analysis was performed across all myeloid cells states. The genes downregulated (statistically significant adjusted p-value <0.05) in the anti-CD14 treated group was used to construct a gene set score and plotted in UMAP space to assess cell states which are deleted in the setting of anti-CD14 treatment.

A dotplot was plotted for this signature to isolate cell states which expressed the highest value. These genes were then used to create a bulk average expression heatmap (row normalized) grouped by anti-CD14, isotype, and saline.

Palantir was used for pseudotime trajectory analysis using Ly6c2 high monocytes as the starting state, number of waypoints as 500 and all other parameters were the default as per Palantir package. All subsequent pseudotime visualization was performed in Python within the Palantir package.

***Single cell (macrophage-specific) and whole tissue proteomics (D3 post-STEMI):*** Whole/bulk cardiac tissue (left ventricular infarcted apex) was lysed in 8 M urea (ThermoFisher Scientific, Grand Island, US), 50 mM HEPES (ThermoFisher Scientific, Grand Island, US) pH 8.0, supplemented with HALT protease and phosphatase inhibitor (78442, ThermoFisher Scientific, Grand Island, US) solubilized by tip-probe sonication on ice. Flow sorted macrophage cell populations were lysed in 6 M urea, with 50 mM HEPES pH 8.0 with HALT protease and phosphatase inhibitor (78442 Pierce; ThermoFisher Scientific, Rockford, US). The protein concentration of all samples was quantified by microBCA (ThermoFisher Scientific, Grand Island, US). A highly sensitive Sera‐Mag-based workflow (magnetic carboxylate modified particles (65152105050250, 45152105050250, Cytiva, Lane Cove West, AU) was employed to streamline enzymatic Lys-C and tryptic digestion of samples (10 μg whole cardiac tissue, 3 μg macrophages). Peptides were quantified by Fluorometric Peptide Assay (23290 ThermoFisher Scientific, Grand Island, US) and samples normalized for immediate analysis. Peptides were analyzed on a Dionex UltiMate NCS-3000RSLC nanoUHPLC (ThermoFisher Scientific, San Jose, US) coupled to high-resolution Q-Exactive HF-X hybrid quadrupole-Orbitrap mass spectrometer equipped with a nanospray ion source (ThermoFisher Scientific, San Jose, US) in data-dependent acquisition analysis over 110 minutes in positive mode. Internal calibration/standards were pre-run to establish instrument performance. Bioinformatic analysis was performed using established highly stringent analysis pipelines for quantitative proteomic data analysis, involving data normalization, quantitation, and differential analysis. Proteomics raw data are available from the ProteomeXchange Consortium via the PRIDE partner repository (http://www.proteomexchange.org/); PXD041192.

Statistical Analysis

All biometric, physiological, histological, and cytometric data were analysed using GraphPad Prism version 9.4.1 (GraphPad Software, San Diego, USA) using one-way analysis of variance (ANOVA) with Tukey's (all pairwise) or Dunnett's (pairwise with baseline) post-hoc test for multiple comparisons, as appropriate to compare STEMI groups only i.e. saline- vs isotype- vs anti-CD14-treated. Normality was assessed for all parameters using the Shapiro-Wilk test. Homogeneity of variance was assessed for all reported parameters using Brown-Forsythe testing, and Kruskal-Wallis (non-parametric) test used where appropriate. After each blinded and randomized trial was reported to the trial sponsor, a cohort of saline-treated sham-operated animals (performed within the same experiments) was analysed post-hoc and included for reader reference only (accordingly omitted from statistical analyses, other than for description of the STEMI model phenotype where comparisons were made between saline-treated sham control and saline-treated STEMI groups using Student’s t-test). All data are presented as mean ± SEM. A p-value of <0.05 was determined to be statistically significant for all datasets.

Circulating cytokine and growth factor levels are presented as median with 25th-75th percentiles (Q1-Q3) and compared using Mann-Whitney U test (2 groups) or Kruskal-Wallis test (>2 groups).

GO enrichment analysis was conducted using the ‘enrichGO’ function within the ‘clusterProfiler’ R package version 4.4.4. Reference genomes obtained from http://geneontology.org were employed for all presented GO analyses. Enrichment analysis specifically for GO Biological Process terms (GO-BP) involved mapping our set of differentially expressed genes (adjusted P value < 0.01) to the *Mus musculus* background gene list. Statistically significant GO-BP terms were identified using a Benjamini-Hochberg adjusted P value cutoff of 0.01. Proteome intensities were processed via VSN normalization method (Limma package version 3.65.3) in R. For proteomics analyses the Human Protein Atlas (https://www.proteinatlas.org/human proteome/tissue) and functional enrichment annotations using g:Profiler (https://biit.cs.ut.ee/gprofiler/) were used. Further pathway enrichment map analysis was performed using Cytoscape (v3.7.1), Reactome, and DAVID functional annotation software. Specifically for macrophage proteome, t-statistic of DE analysis was subjected to 1D enrichment analysis in Perseus software for functional enrichment of Gene Ontology Biological Processes (GOBP), Cellular components (GOCC) and Molecular Functions (GOMF). For cardiac apex proteome, differentially regulated proteins (p< 0.05) were used for functional enrichment analysis in Cytoscape StringApp focusing on gene ontology (GOBP, GOCC, GOMF) and pathways (KEGG, Reactome, Wikipathway). Data visualizations were completed using either Cytoscape or R (ggplot2 package).

Upon completion of DE analysis for single-cell RNA sequencing and proteomics, a comparison of DE genes was performed using R packages, including RVenn (v1.1.0), VennDiagram (v1.7.3), and Venneuler (v1.1-3). Differentially expressed genes from all datasets were filtered based on P value threshold (P<0.01). Common differentially expressed genes were determined using the ‘overlap’ function within RVenn. Subsequently, Gene Ontology (GO) analysis was conducted on each overlapping gene list derived from this analysis, elucidating biologically relevant pathways specific to each set of overlapping genes.

## References for online methods and supplementary material

1. Lo, M., et al., *Effector-attenuating Substitutions That Maintain Antibody Stability and Reduce Toxicity in Mice.* J Biol Chem, 2017. **292**(9): p. 3900-3908.

2. Chen, J., et al., *Variability in coronary artery anatomy affects consistency of cardiac damage after myocardial infarction in mice.* Am J Physiol Heart Circ Physiol, 2017. **313**(2): p. H275-H282.

3. Lindsey, M.L., et al., *Reperfused vs. nonreperfused myocardial infarction: when to use which model.* Am J Physiol Heart Circ Physiol, 2021. **321**(1): p. H208-H213.

4. Xin, A., et al., *Significance of QRS scoring system in left ventricular function recovery after acute myocardial infarction.* ESC Heart Fail, 2024. **11**(5): p. 2778-2788.

5. Gaudron, P., et al., *Progressive left ventricular dysfunction and remodeling after myocardial infarction. Potential mechanisms and early predictors.* Circulation, 1993. **87**(3): p. 755-63.

6. Weeks, K.L., et al., *CORP: Practical tools for improving experimental design and reporting of laboratory studies of cardiovascular physiology and metabolism.* Am J Physiol Heart Circ Physiol, 2019. **317**(3): p. H627-H639.

7. Donner, D.G., et al., *Improving the quality of preclinical research echocardiography: observations, training, and guidelines for measurement.* Am J Physiol Heart Circ Physiol, 2018. **315**(1): p. H58-H70.

8. Tao, B., et al., *Preclinical modeling and multimodality imaging of chronic myocardial infarction in minipigs induced by novel interventional embolization technique*, in *EJNMMI Research*. 2016, Springer Berlin Heidelberg. p. 59.

9. Frantz, S., et al., *Left ventricular remodelling post-myocardial infarction: pathophysiology, imaging, and novel therapies.* Eur Heart J, 2022. **43**(27): p. 2549-2561.

10. Flachskampf, F.A., et al., *Cardiac imaging after myocardial infarction*, in *European Heart Journal*. 2011, Oxford University Press. p. 272-283.

# SUPPLEMENTARY RESULTS

## Safety Profile of CD14 Blockade with Anti-CD14 Antibody

No adverse events were observed following anti-CD14 treatment commencement (**Supplementary Table S1**). Left atrial thrombus was observed in one mouse in the isotype-treated group only (7-days post-STEMI cohort).

## Model of Reperfused Anterior STEMI Develops Progressive LV Systolic Dysfunction, Remodeling, and Hemodynamic Decompensation following STEMI

Following 1 h of left anterior descending coronary artery occlusion and closed chest reperfusion, hearts had an AAR comprising approximately 50 % of the total LV (see **Supplementary Figure S3B-D**), and hearts of saline-treated control mice developed an acute infarct size of 65 ± 4 (% of AAR) measured by planimetric analysis of serially sectioned and dual-stained (by EB/TTC) whole hearts excised 24 h post-STEMI (**Supplementary Figure S3C**. At 24 h post-STEMI, untreated mice exhibited depressed LV systolic function (29±2 % EF) with evidence of early adverse remodeling i.e. increased end-diastolic volume (74±3 µl EDV), observed by ultra-high frequency parasternal long axis echocardiography (**Supplementary Figure S3E-I**), partially associated with acute myocardial stunning.

At 3 days post-STEMI, untreated mice presented with similar LV systolic dysfunction (31±1 % EF) and dilatation (74±3 µl EDV, **Supplementary Figure S3J-N**).

At 7 days post-STEMI without treatment, LV dilatation remained stable (77±3 vs 74±3 µl EDV at D3 post-STEMI, p=0.55). However, systolic dysfunction progressively worsened without treatment (25±1 vs 31±1% EF at D3 post-STEMI, p<0.001) alongside reduced LV stroke volume (19±1 vs 23±1 µl at D3, p<0.01) and cardiac output (9.9±0.4 vs 12.5±0.5 ml/min at D3, p<0.001) (**Figure 2B**). Invasive hemodynamics were similarly impacted at 7 days post-STEMI, including significantly reduced developed pressure (i.e. delta between end-systolic end-diastolic LV pressure; 78±2 vs 92±3 mmHg in shams, p<0.05), and approximately halved LV stroke work (1,103±63 vs 2,075±309 mmHg x µl in shams, p<0.001), assessed by LV pressure-volume (PV) catheterization (**Figure 2C**).

At 21 days post-STEMI, cardiac magnetic resonance (CMR) imaging provided an additional assessment of LV volumes, further validating echocardiographic observations of LV dilatation without treatment (i.e. 102±8 µl in saline controls). At 28 days, echocardiography showed that LV systolic dysfunction had remained depressed but stable compared with day 7 (24±1 vs 25±2% EF at D7 post-STEMI, p=0.56), alongside progressive LV dilatation (111±6 vs 77±3 µl EDV at D7 post-STEMI, p<0.001, **Figure 2F**). Global longitudinal strain (GLS) of the LV was also suppressed at this timepoint (-6.8±0.3 vs -12.6±0.7 % in shams, p<0.001). As observed at day 7 post-STEMI, LV developed pressure remained depressed at 28 days (80±2 vs 91±2 mmHg in shams, p<0.01) with the additional development of reduced +dP/dT (6,902±248 vs 9,446±497 in shams, p<0.01) and -dP/dT (-5,928±476 vs -8,737±443 in shams, p<0.01) at this timepoint (**Figure 2G**).

Together these changes are reflected in a shrinking and overt rightward shift of the PV loop between days 7 and 28 in this model of murine STEMI (**Figure 2**). In our extensive experience with this model, no significant additive functional decompensation or remodeling is observed beyond 28 days post-STEMI.

# SUPPLEMENTARY TABLES AND FIGURES

| **Adverse Events** | **Saline**  (n=76) | **Isotype**  (n=66) | **Anti-CD14**  (n=102) |
| --- | --- | --- | --- |
| Atrial thrombus (%) | 0 (0%) | 1 (1.5%) | 0 (0%) |
| Cardiac rupture (%) | 0 (0%) | 0 (0%) | 0 (0%) |
| Lung congestion (%) | 0 (0%) | 0 (0%) | 0 (0%) |
| Aneurism (%) | 0 (0%) | 0 (0%) | 0 (0%) |
| Evidence of infection (%) | 0 (0%) | 0 (0%) | 0 (0%) |
| Death or emergency euthanasia (%) | 0 (0%) | 0 (0%) | 0 (0%) |

## Supplementary Table S1. Safety and Adverse Events. Including all observations at post-mortem in animals that survived 1 h ischemia and received any treatment by intravenous injection at time of reperfusion.

| **Stain or Antibody** | **Supplier (Cat #)** |
| --- | --- |
| Masson’s trichrome | Sigma-Aldrich, Stenheim, DE (HT15) |
| Picrosirius red | Sigma-Aldrich, Stenheim, DE (2610-10-8) |
| Anti-CD68 | Abcam, Waltham, Waltham, US (Ab125212) |
| Anti-troponin T | ThermoFisher Scientific, Grand Island, US (MA5-12960) |
| DAPI, hydrochloride | ThermoFisher Scientific, Grand Island, US (D1306) |

**Supplementary Table S2.** **(Immuno)histochemical approach and reagents**

| **Cell type** | **Antibody signature (staining pattern)** |
| --- | --- |
| Viable Leukocytes | CD45+ |
| Granulocytes | CD45+, CD11b+, Ly6G+ |
| Macrophages | CD45+, CD11b+, CD64+ |
| CD14+ Macrophages | CD45+, CD11b+, CD64+, CD14+ |
| CD14- Macrophages | CD45+, CD11b+, CD64+, CD14- |
| MHCII-hi Macrophages | CD45+, CD11b+, CD64+, MHCII+ |
| MHCII-hi Mrc1+ Macrophages | CD45+, CD11b+, CD64+, MHCII+, MRC1+ |
| MHCII-low; Mrc1+ Macrophages | CD45+, CD11b+, CD64+, MHCII-, MRC1+ |
| MHCII-low Macrophages | CD45+, CD11b+, CD64+, MHCII- |
| Myeloids | CD45+, CD11b+, Ly6G- |
| Non-myeloids | CD45+, CD11b-, Ly6G- |

**Supplementary Table S3. Leukocyte subtype staining patterns**

| ***Flow cytometry*** | | | | | |
| --- | --- | --- | --- | --- | --- |
| **Antibody Target** | **Dye** | **Cat#** | **Company** | **Clone** | **Final Dilution** |
| CD45 | APC-Cy7 | 561037 | BD Biosciences, San Jose, US | 30-F11 | 1:400 |
| I-A/I-E (MHCII) | OptiBuild BUV395 | 743876 | BD Biosciences | 2G9 | 1:400 |
| CD11b | Horizon BUV737 | 612801 | BD Biosciences | M1/70 | 1:400 |
| CD14 | Brilliant violet | 123337 | Biolegend, San Diego, US | Sa14-2 | 1:200 |
| Ly-6C | FITC | 128005 | Biolegend | HK1.4 | 1:400 |
| Ly-6G | PE/dazzle | 127647 | Biolegend | 1A8 | 1:200 |
| CD64 (a & b alloantigens) | PE/cyanine7 | 139313 | Biolegend | X54- 5/7.1 | 1:200 |
| CD206 (Mrc1) | AF647 | 141711 | Biolegend | C068C2 | 1:200 |
| N/A (nucleus) | DAPI (1 mg/mL) | D9542 | Sigma Aldrich | N/A | 1:1000 |
| ***scRNA-seq*** | | | | | |
| **Antibody Target** | **Dye** | **Cat#** | **Company** | **Clone** | **Final Dilution** |
| CD45 | APC-Cy7 | 561037 | BD Biosciences | 30-F11 | 1:400 |
| CD11b | Horizon BUV737 | 612801 | BD Biosciences | M1/70 | 1:400 |
| CD64 | PE/cyanine7 | 139313 | Biolegend | X54- 5/7.1 | 1:200 |
| N/A (nucleus) | DAPI (1 mg/mL) | D9542 | Sigma Aldrich | N/A | 1:1000 |

**Supplementary Table S4. Antibodies used in Flow Cytometry and scRNAseq experiments.**

| **Cytokine** | **Day 1 post-STEMI** | | | | **Day 3 post-STEMI** | | | | **Day 1 vs Day 3** | | |
| --- | --- | --- | --- | --- | --- | --- | --- | --- | --- | --- | --- |
|  | Saline  (n=10) | Isotype  (n=12) | Anti-CD14  (n=11) | p-value | Saline  (n=20) | Isotype  (n=20) | Anti-CD14  (n=20) | p-value | Saline  p-value | Isotype  p-value | Anti-CD14  p-value |
| Eotaxin | 441.86 (417.87-585.74) | 472.42 (412.23-548.58) | 464.41 (432.69-543.11) | 0.95 | 561.11 (465.12-623.31) | 584.26 (494.99-649.69) | 534.07 (478.73-600.95) | 0.57 | 0.47 | 0.063 | 0.28 |
| G-CSF | 785.92 (623.33-1099.86) | 768.05 (560.61-996.63) | 711.39 (594.12-945.79) | 0.96 | 319.96 (280.25-424.99) | 315.3 (258.52-408.36) | 307.32 (247.85-402.96) | 0.65 | **<0.001** | **<0.001** | **0.003** |
| GM-CSF | 31.42 (26.34-39.88) | 40.78 (27.77-46.95) | 31.37 (24.21-43.22) | 0.23 | 53.07 (50.56-54.98) | 53.07 (51.16-54.33) | 52.03 (50.02-54.02) | 0.71 | **<0.001** | **<0.001** | **<0.001** |
| IFNγ | 4.3 (3.86-7.26) | 6.63 (3.96-8.59) | 4.89 (3.51-7.08) | 0.40 | 10.99 (9.72-11.38) | 10.8 (10.07-12.18) | 10.57 (9.77-11.56) | 0.85 | **<0.001** | **<0.001** | **<0.001** |
| IL-1α | 312.12 (271.34-404.27) | 375.78 (301.31-396.96) | 328.44 (262.47-395.71) | 0.70 | 436.39 (415.04-455.32) | 451.43 (433.48-471.39) | 434 (414.34-461.21) | 0.21 | **<0.001** | **<0.001** | **<0.001** |
| IL-1β | 21.46 (19.07-32.89) | 32.2 (20.85-40.71) | 25.48 (18.15-33.24) | 0.43 | 47.85 (45.98-51.22) | 49.21 (47.17-52.25) | 46.99 (45.05-49.36) | 0.16 | **<0.001** | **<0.001** | **<0.001** |
| IL-2 | 56.53 (51.54-74.45) | 69.29 (53.09-77.67) | 64.62 (51.15-73.23) | 0.71 | 88.74 (85.62-90.71) | 88.68 (86.93-91.43) | 87.25 (86.11-90.52) | 0.58 | **<0.001** | **<0.001** | **<0.001** |
| IL-3 | 4.44 (3.81-6.74) | 6.8 (3.79-7.9) | 4.47 (3.6-6.79) | 0.29 | 10.09 (9.49-10.51) | 10.11 (9.19-10.51) | 10.12 (9.45-10.36) | 0.97 | **<0.001** | **<0.001** | **<0.001** |
| IL-4 | 2.11 (1.65-3.05) | 3.24 (1.91-3.61) | 2.28 (1.63-3.18) | 0.16 | 3.94 (3.88-4.09) | 4 (3.82-4.17) | 3.9 (3.79-4.15) | 0.78 | **0.001** | **<0.001** | **<0.001** |
| IL-5 | 9.8 (7.2-13.47) | 12.24 (10.13-15.95) | 8.92 (8.09-11.44) | 0.059 | 24.01 (17.61-32.65) | 21.72 (14.45-34.23) | 27.94 (21.07-42.28) | 0.37 | **<0.001** | **0.010** | **<0.001** |
| IL-6 | 37.72 (18.42-81.49) | 42.02 (32.59-49.45) | 39.02 (18.79-114.57) | 0.98 | 40.53 (22.97-56) | 30.29 (19.98-56.05) | 32.31 (24.26-66) | 0.61 | 0.99 | 0.91 | 0.97 |
| IL-7 | 12.31 (7.78-17.95) | 15.88 (8.13-20.13) | 15.14 (9.93-17.3) | 0.66 | 26.41 (23.83-28.65) | 26.67 (25.71-28.21) | 26.54 (25.47-30.09) | 0.76 | **<0.001** | **<0.001** | **<0.001** |
| IL-9 | 78.47 (73.4-105.07) | 117.48 (65.89-123.11) | 106.01 (68.54-122.23) | 0.82 | 142.86 (137.88-149.36) | 144.59 (138.02-151.28) | 141.79 (136.45-148.89) | 0.72 | **<0.001** | **<0.001** | **<0.001** |
| IL-10 | 21.35 (16.35-31.2) | 29.56 (17.4-41.26) | 23.95 (15.29-33.27) | 0.45 | 44.37 (42.01-48.09) | 45.49 (42.85-47.69) | 44.81 (41.51-47.2) | 0.43 | **<0.001** | **<0.001** | **<0.001** |
| IL-12p40 | 39.36 (35.78-63.87) | 56.73 (34.78-75.92) | 47.68 (33.48-60.34) | 0.43 | 84.82 (81-90.38) | 86.61 (81.62-92.58) | 81.35 (77.6-90.66) | 0.53 | **<0.001** | **<0.001** | **<0.001** |
| IL-12p70 | 123.92 (104.03-170.74) | 158.22 (121.12-186.97) | 126.27 (104.71-170.51) | 0.44 | 220.91 (210.49-232.8) | 219.22 (213.52-227.31) | 214.07 (205.49-221.9) | 0.35 | **<0.001** | **<0.001** | **<0.001** |
| IL-13 | 58.13 (48.14-77.92) | 71.79 (51.44-84.16) | 62.85 (51.56-75.16) | 0.52 | 102.34 (97.66-105.96) | 100.06 (96.16-104.69) | 98.86 (94.25-100.48) | 0.28 | **<0.001** | **<0.001** | **<0.001** |
| IL-15 | 150.97 (122.38-182.35) | 172.4 (115.34-207.99) | 185.66 (149.7-237.05) | 0.25 | 247.56 (238.01-261.26) | 258.07 (245.49-280.4) | 271.79 (251.38-281.36) | 0.088 | **<0.001** | **<0.001** | **0.002** |
| IL-17 | 2.37 (2-3.59) | 3.45 (2.07-4.1) | 2.64 (1.87-3.72) | 0.48 | 5.04 (4.57-5.19) | 5.02 (4.73-5.1) | 4.71 (4.53-4.98) | 0.085 | **<0.001** | **<0.001** | **<0.001** |
| IP-10 | 26.53 (21.75-31.34) | 29.29 (25.15-35.02) | 27.81 (22.23-34.52) | 0.43 | 27.16 (25.52-31.09) | 28.55 (24.67-32.78) | 29.37 (27.26-33.13) | 0.57 | 0.82 | 0.91 | 0.97 |
| KC | 150.22 (121.55-302.35) | 168.09 (117.51-210.73) | 120.22 (102.27-181.92) | 0.69 | 185.48 (132.38-306.53) | 149.81 (121.72-190.76) | 178.51 (115.63-264.11) | 0.35 | 0.85 | 0.95 | 0.85 |
| LIF | 4.21 (3.31-5.66) | 4.9 (2.99-6.46) | 5.05 (3.87-5.98) | 0.79 | 7.65 (7.22-7.8) | 7.99 (7.52-8.47) | 7.76 (7.41-8.12) | 0.28 | **<0.001** | **<0.001** | **<0.001** |
| LIX | 348.07 (230.2-924.41) | 582.33 (169.94-1102.93) | 398.24 (207.68-1047.17) | 0.98 | 278.43 (226.66-377.98) | 323 (214.93-590.62) | 319.71 (233.55-447.98) | 0.74 | 0.82 | 0.91 | 0.87 |
| MCP-1 | 131.7 (115.11-147.56) | 150.22 (117.9-171.54) | 126.99 (116.19-161.91) | 0.38 | 183.06 (175.08-187.48) | 182.42 (172.95-192.35) | 183.38 (172.37-195.57) | 0.85 | **<0.001** | **0.002** | **<0.001** |
| M-CSF | 48.63 (40.01-75.08) | 64.62 (43.18-87.57) | 52.72 (40.71-70.71) | 0.48 | 96.87 (92.66-101.55) | 96.74 (92.45-101.09) | 93.67 (89.89-100.51) | 0.49 | **<0.001** | **0.005** | **<0.001** |
| MIG | 150.19 (42.4-308.31) | 148.77 (47.77-416.27) | 128.46 (41.81-240.62) | 0.85 | 62.76 (50.41-138.07) | 82.44 (62.6-129.66) | 94.67 (60.64-151.14) | 0.35 | 0.85 | 0.86 | 0.97 |
| MIP-1α | 105.63 (89.16-123.91) | 115.44 (104.59-124.32) | 97.76 (89.66-119.51) | 0.22 | 128.52 (122.73-134.21) | 129.5 (126.16-132.69) | 129.68 (124.78-132.38) | 0.88 | **0.018** | **0.005** | **<0.001** |
| MIP-1β | 107.29 (96.8-116.2) | 120.44 (109.01-140.75) | 102.56 (91.24-124.49) | 0.13 | 132.67 (127.16-137.48) | 133.28 (129.28-143.74) | 139.43 (127.83-143.89) | 0.40 | **<0.001** | 0.28 | **0.001** |
| MIP-2 | 315.87 (292.56-400.26) | 398.1 (298.77-431.24) | 342.46 (291.89-399.89) | 0.44 | 469.91 (459.66-485.63) | 469.27 (453.82-482.34) | 469.29 (450.64-479.18) | 0.68 | **<0.001** | **<0.001** | **<0.001** |
| RANTES | 21.24 (18.3-26.54) | 26.44 (20.57-30.92) | 25.59 (19.86-29.89) | 0.49 | 35.91 (34.83-38.51) | 36.37 (34.34-37.99) | 36.27 (35.11-38) | 0.95 | **<0.001** | **<0.001** | **<0.001** |
| TNFα | 19.81 (17.83-26.87) | 26.44 (19.23-31.13) | 20.73 (18.89-28.8) | 0.21 | 33.5 (31.96-35.34) | 34.17 (32.89-35.28) | 33.02 (31.79-34.23) | 0.16 | **<0.001** | **<0.001** | **<0.001** |
| VEGF | 1.41 (1.17-1.92) | 1.83 (1.27-2.13) | 1.36 (1.14-1.86) | 0.24 | 2.41 (2.28-2.53) | 2.46 (2.37-2.53) | 2.44 (2.31-2.56) | 0.80 | **<0.001** | **<0.001** | **<0.001** |

**Supplementary Table S5. Circulating growth factor and cytokine levels following CD14 Blockade at D1 and D3 post-STEMI.** Biomarker concentrations in pg/ml. Data presented as median (Q1-Q3), with Mann-Whitney U-test conducted between D1 and D3 and Kruskal Wallis test to compare between the three groups at each timepoint.


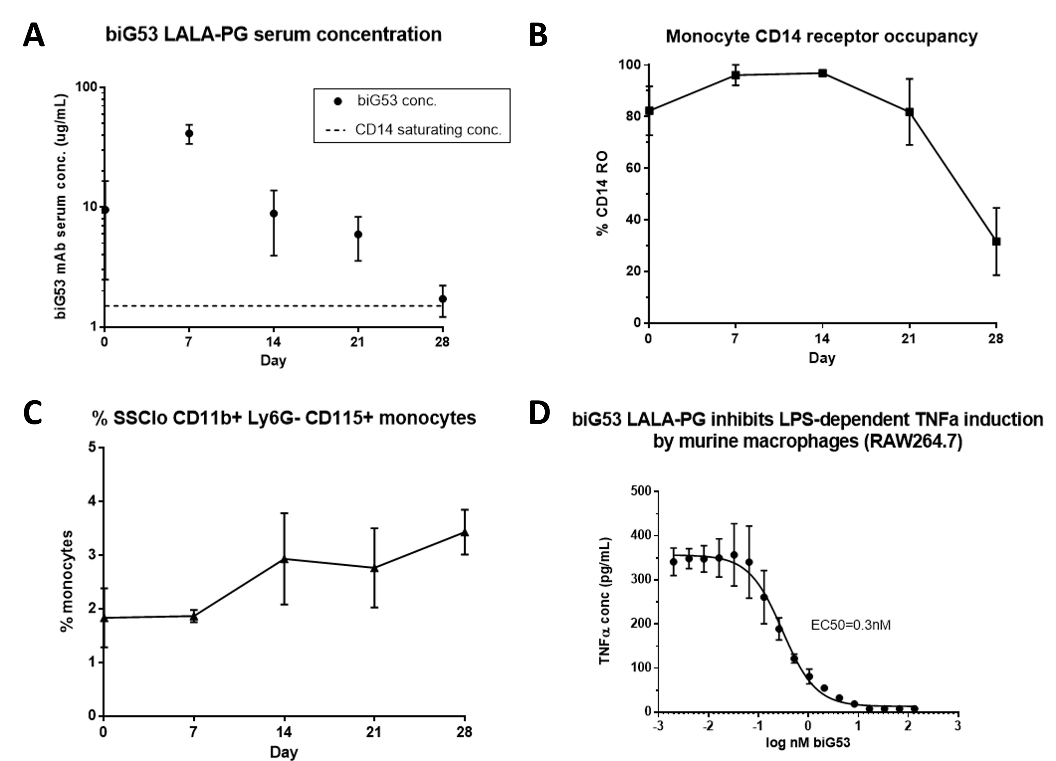


## Supplementary Figure S1. Anti-CD14 serum PK, CD14 receptor occupancy, and monocyte abundance following a 5 mg/kg anti-CD14 mAb infusion. C57BL6 mice (n=3) received a 5 mg/kg infusion of biG53 LALA-PG mAb (anti-CD14) at day 0, with serum and whole blood collection at 0.5 h, 7-, 14-, 21-, and 28-days post-injection. WinNonlin analysis of anti-CD14 serum levels determined a terminal antibody half-life of 160 h. A) A single 5 mg/kg infusion of anti-CD14 achieves serum concentrations necessary for monocyte CD14 saturation (i.e. 1.5 ug/mL) for ~28 days. *In vitro* titration of anti-CD14 on C57BL6 whole blood, followed by monocyte CD14 receptor occupancy analysis by flow cytometry demonstrated monocyte CD14 saturation is reached at 1.5 ug/mL (dashed line). B) Monocyte CD14 receptor occupancy as determined by flow cytometry was 96% over 14 days post-infusion. C) A 5 mg/kg infusion of anti-CD14 does not deplete the SSC lo, CD11b+, Ly6G-, CD115+ population as determined by flow cytometry. Graphed values show the mean and standard deviation. D) anti-CD14 blocks LPS-dependent TNF-α secretion in vitro by RAW264.7 cells stimulated with 10 ng/mL LPS. Complete inhibition of TNF-α secretion is achieved at anti-CD14 concentration of 1.5 ug/mL (i.e. 10 nM), the antibody concentration that saturates monocyte CD14 in whole blood. Mean ± SEM.


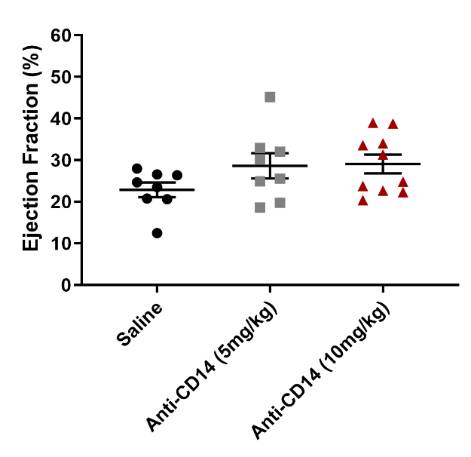

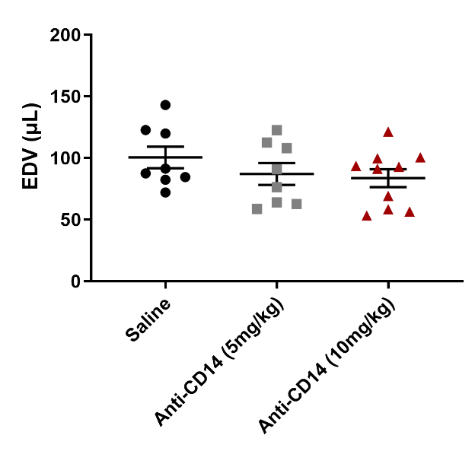


**Supplementary Figure S2. Echocardiographic results of drug dose comparison pilot study measured 7 days post-STEMI.** A small pilot study was designed to confirm systolic function (ejection fraction) effect signal; and determine effect size, standard deviation, and lowest effective dose of anti-CD14 antibody treatment delivered using the same protocols used in the main study i.e. administered intravenously at reperfusion, following 1 h occlusion of the left ventricular left anterior descending coronary artery. EDV – end-diastolic volume. No statistical comparisons were performed in this study. Mean ± SEM.

**D**


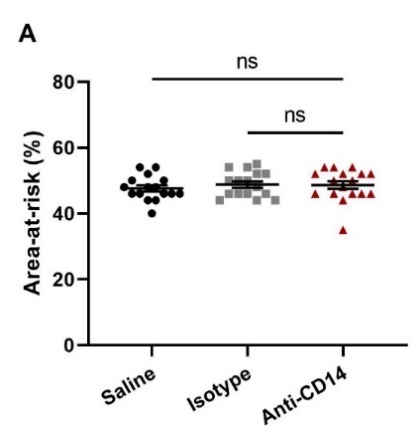

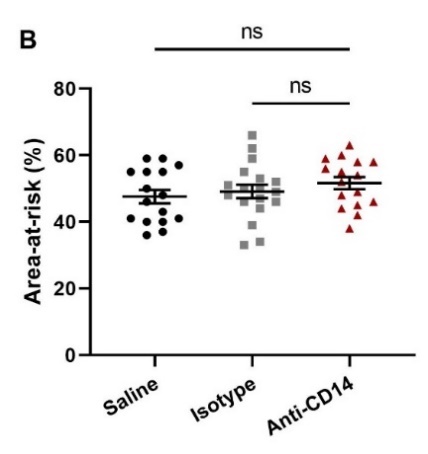

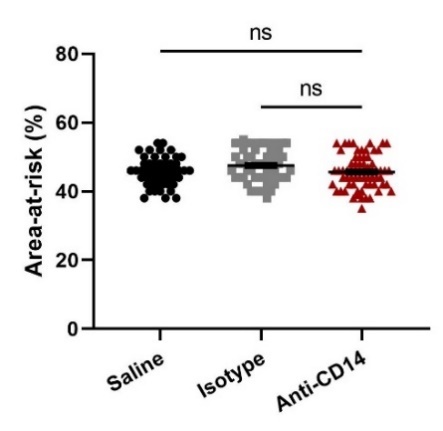

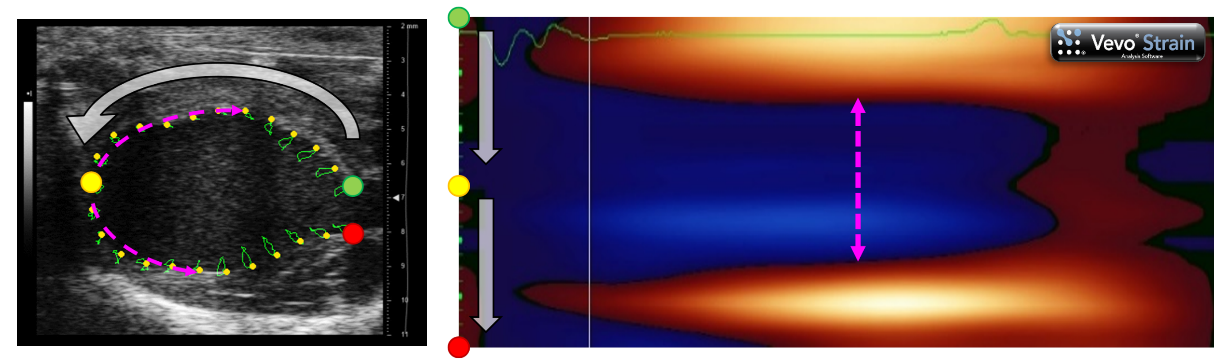


**C**

**B**

**D**

**A**


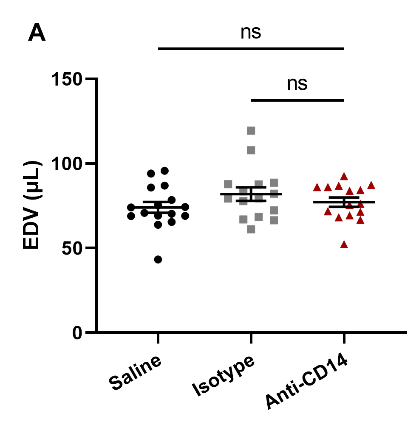

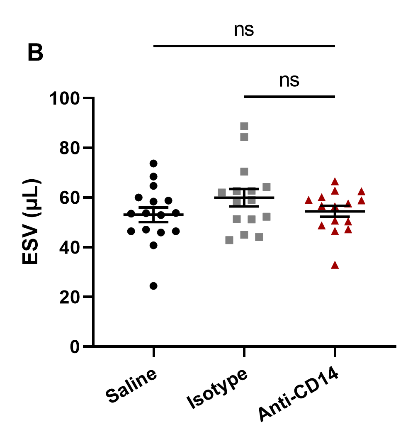

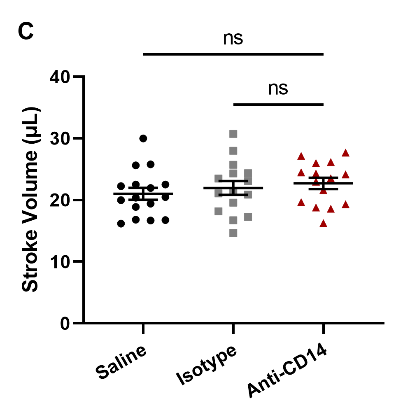


**G**

**F**

**E**


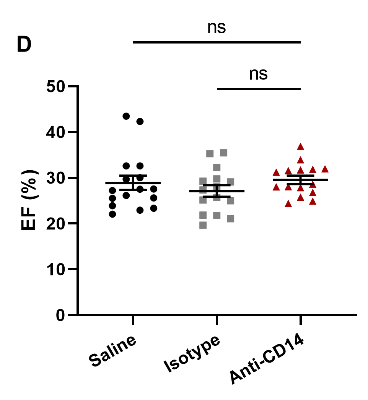

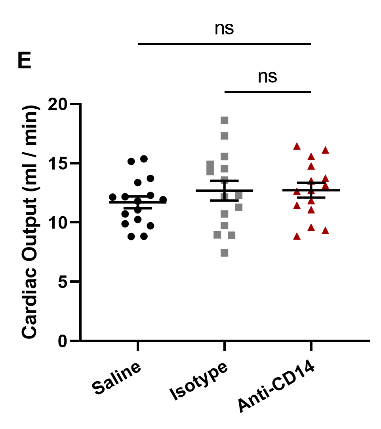


**I**

**H**


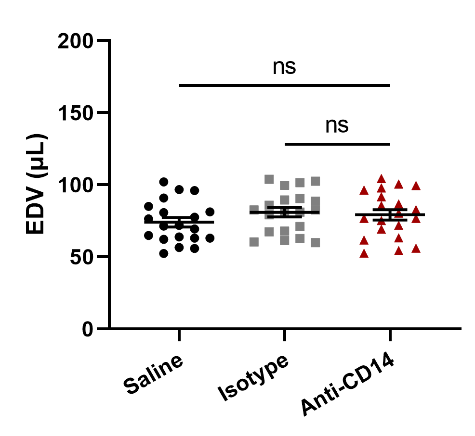

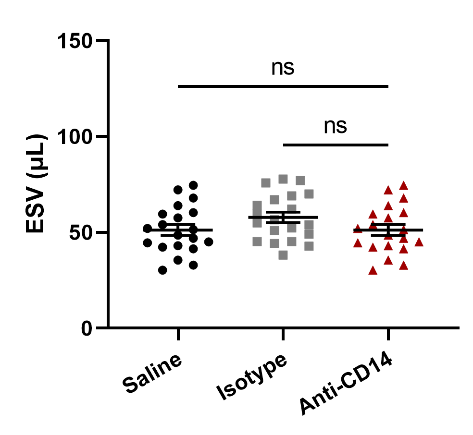

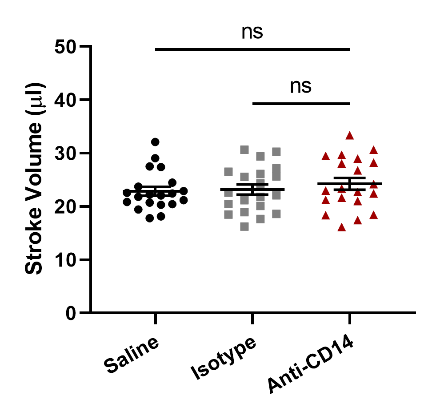

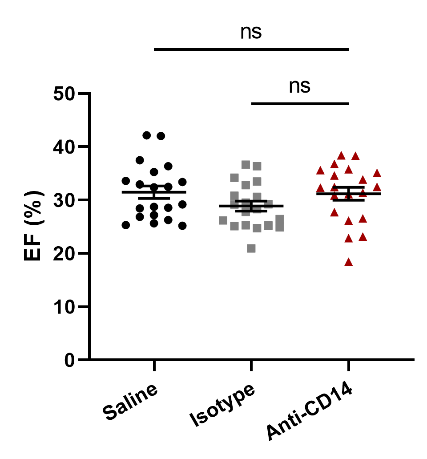

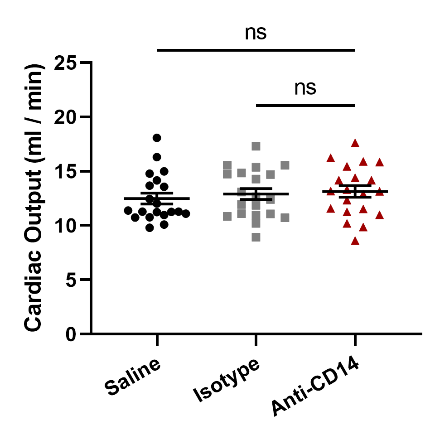


**L**

**N**

**M**

**J**

**K**

**Supplementary Figure S3.** **Echocardiographic assessments at 24 hours and 3 days post-STEMI.** A) Relative wall displacement mapping method for assessing area-at-risk (AAR). The endocardial circumference was traced to track (left) and map (right) relative tissue displacement in a single cardiac cycle (note EKG overlay, green). Colored circles at the endocardium in B-mode correlate with those fit to the y-axis on the displacement map. The pink line denotes inactive/negative endocardial wall displacement (blue area in map). Left Ventricular AAR at 24 h post-STEMI measured by B) *in vivo* echocardiography, and C) planimetric assessment following post-mortem excision and EB/TTC dual staining of the same hearts. D) Left Ventricular Area-at-risk (AAR) at 24 h post-STEMI across all studies, measured by *in vivo* echocardiography, demonstrating consistency of AAR between groups across all studies. Further echocardiographic assessments at 24 h post-STEMI included left ventricular volumes (E-G), systolic function (H), and cardic output (I). Echocardiographic assessments at 3 days post-STEMI included left ventricular volumes (J-L), systolic function (M), and cardic output (N). EDV – end-diastolic volume, ESV – end-systolic volume, EF – ejection fraction. Data were compared using one-way ANOVA with Tukey multiple comparisons post-hoc tests. Mean ± SEM. ns – not significant.


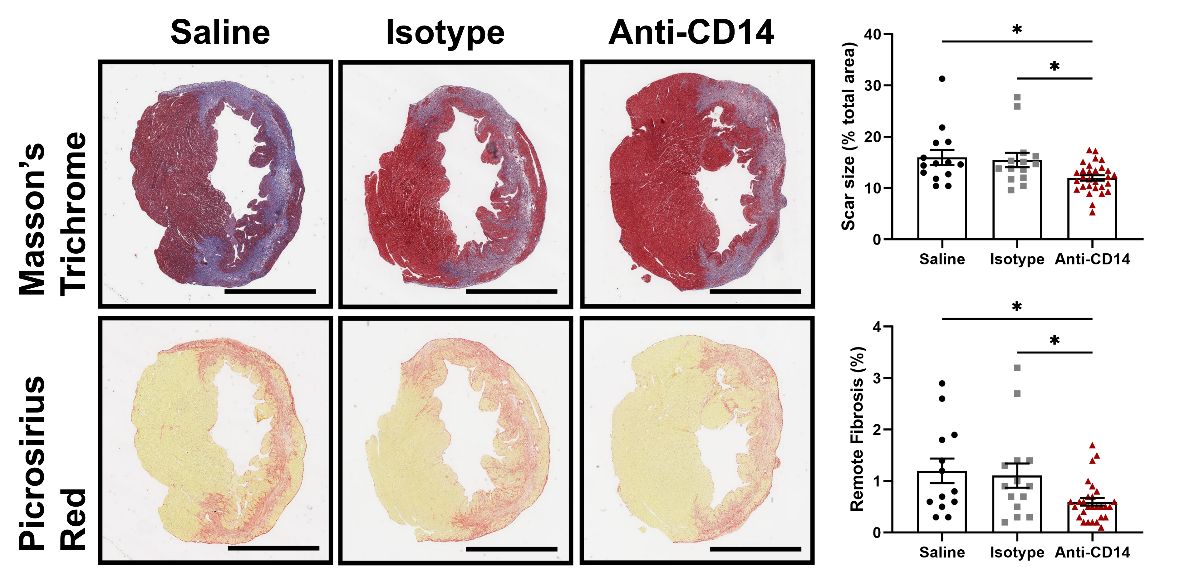


**Supplementary Figure S4. Histological assessment of fibrosis at 7- days post-STEMI.** Representative histological images of Masson’s trichrome (upper panels) and picrosirius red (lower panels, bars 500 µm) at 7 days post-STEMI, and assessments of scar size (free wall lesion percentage of total area) and remote (non-lesion/septal) fibrosis. Data were compared using one-way ANOVA with Tukey multiple comparisons post-hoc tests. Mean ± SEM. *p<0.05, **p<0.01.


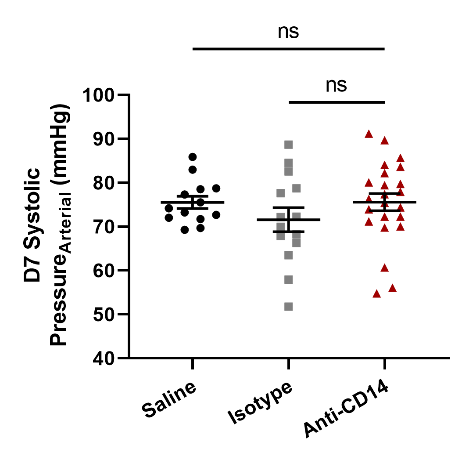

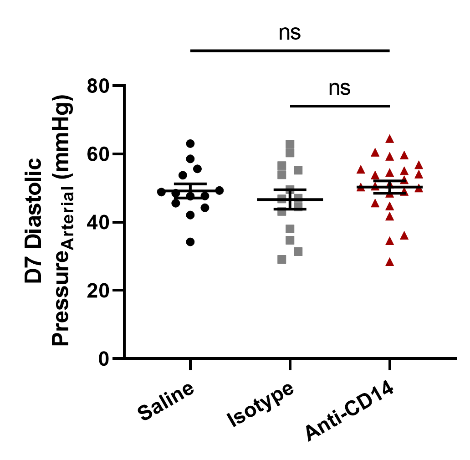


**B**

**A**


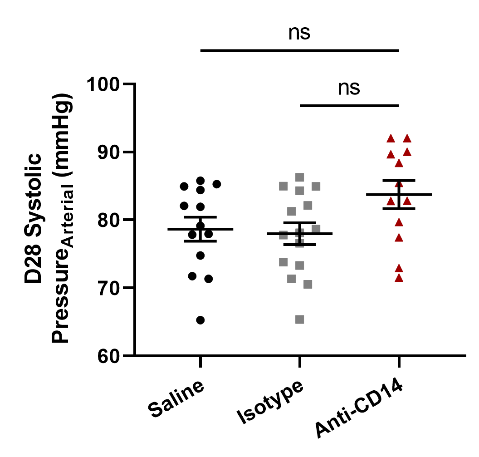

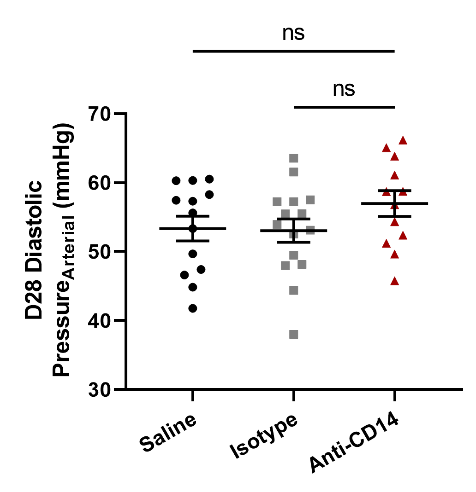


**D**

**C**

## Supplementary Figure S5. Invasive arterial systolic and diastolic pressures measured at either 7 days (D7, A and B) or 28 days (D28, C and D) post-STEMI during pressure-volume (PV) catheterization. Data were compared using one-way ANOVA with Tukey multiple comparisons post-hoc tests. Mean ± SEM. ns – not significant.


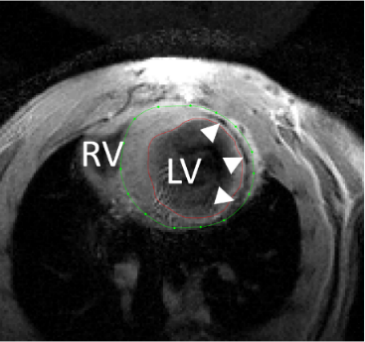


**Supplementary Figure S6. Cardiac magnetic resonance (CMR) imaging analysis example.** Endocardial borders of the left ventricle (LV) are traced at end-diastole and end-systole to determine fractional change (ejection fraction (EF%)). Epicardial borders are traced to define LV wall area (arrows). Note: After 21 days post-STEMI in this model, severe left ventricular free-wall thinning precludes accurate measurement of expanded infarct size.


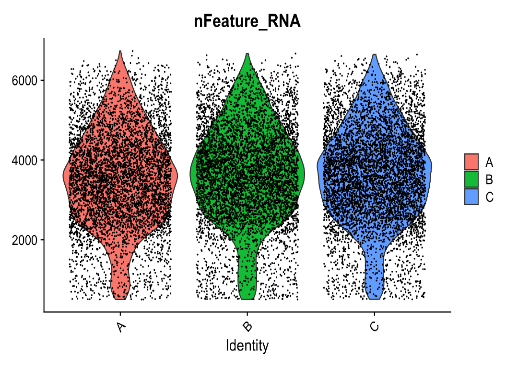

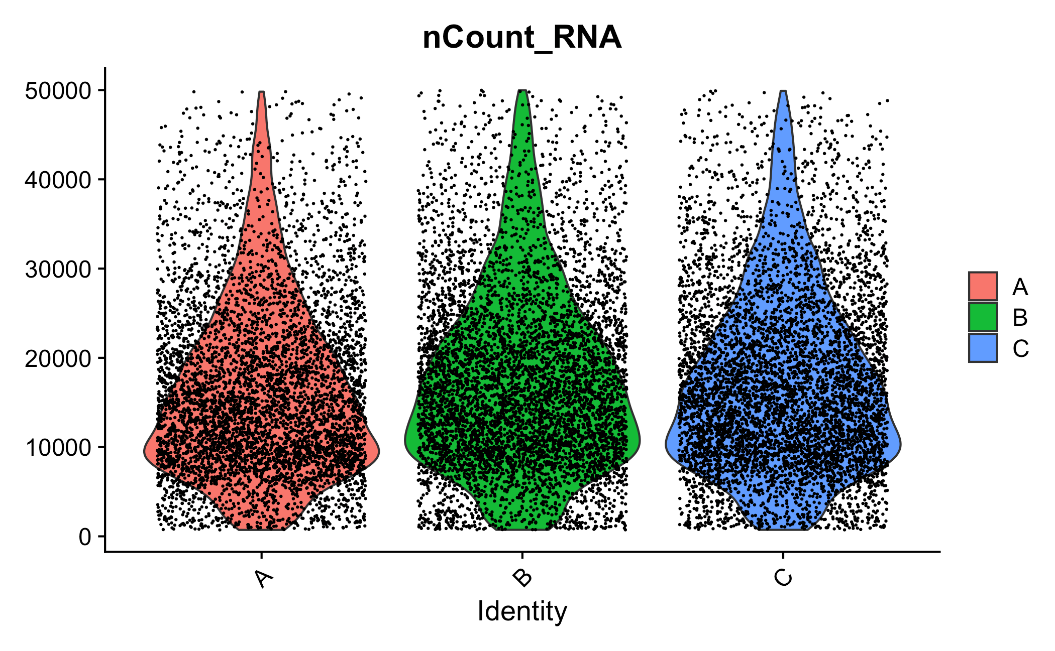

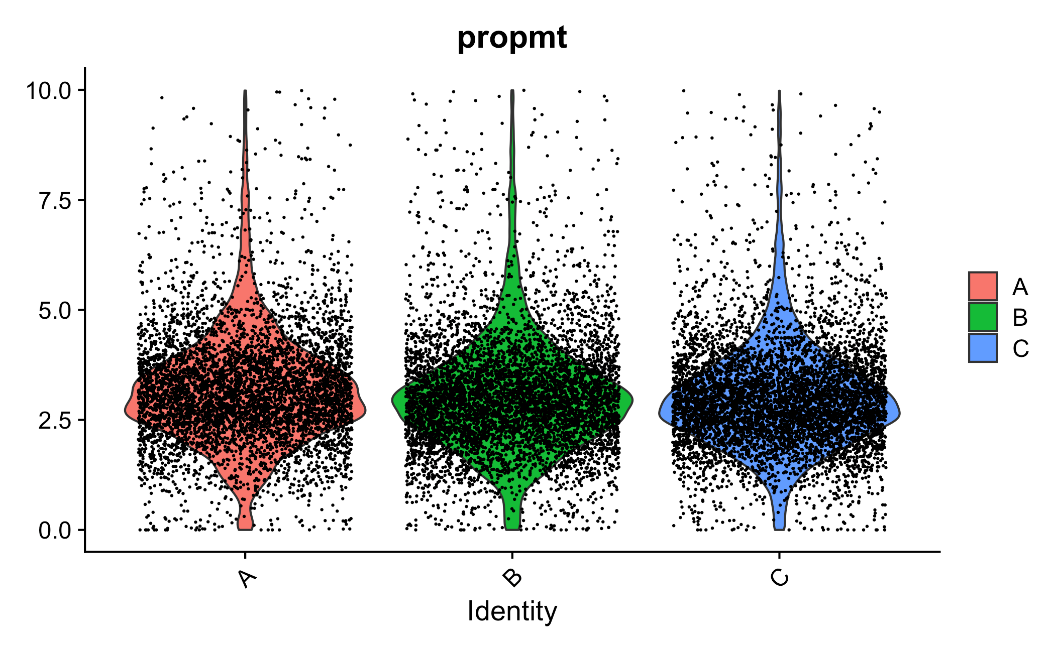


**A**

**B**


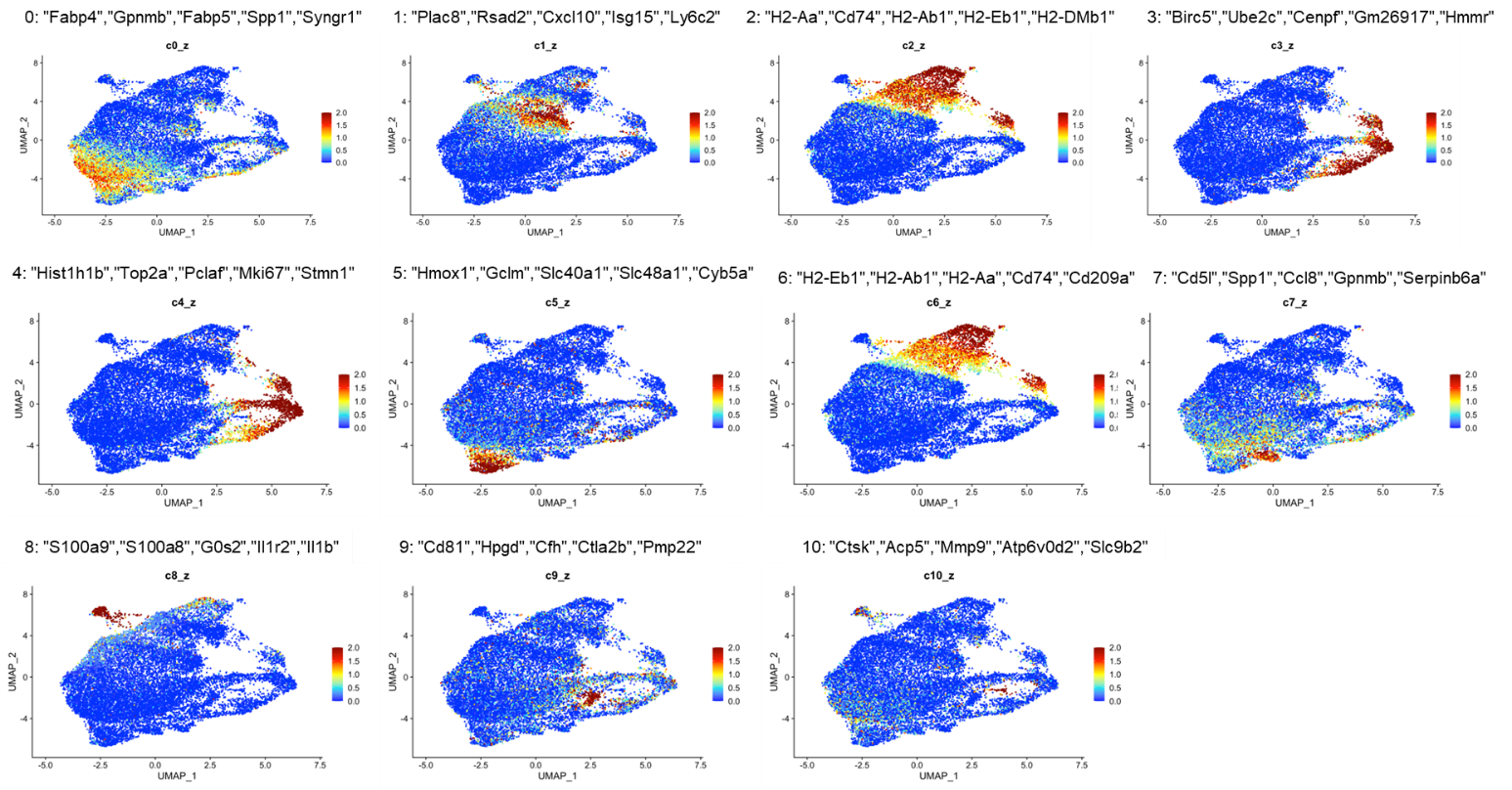


**Supplementary Figure S7. Assessments of scRNAseq data quality.** A**)** Quality control assessments performed on blinded/de-identified groups (A – isotype, B – Anti-CD14, C – Saline). B) Marker gene set z-scores for myeloid cell states (Clusters).


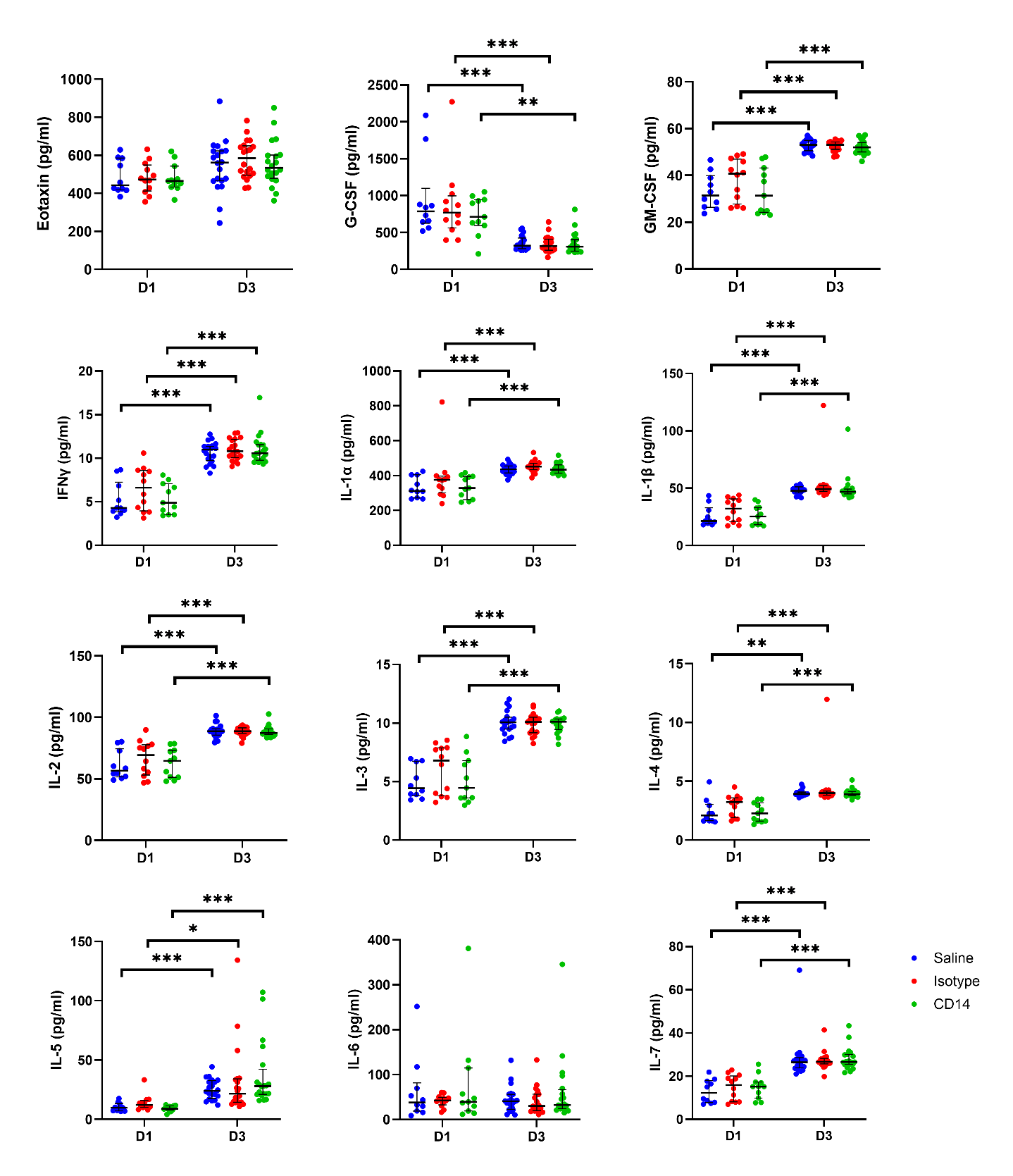


## Supplementary Figure S8. Circulating Growth Factor and cytokine levels following CD14 Blockade at D1 and D3 post-STEMI. Data presented as median (Q1-Q3), with Mann-Whitney U-test conducted between D1 and D3 and Kruskal Wallis test to compare between the three groups at each timepoint. * p<0.05, ** p<0.01, *** p<0.001


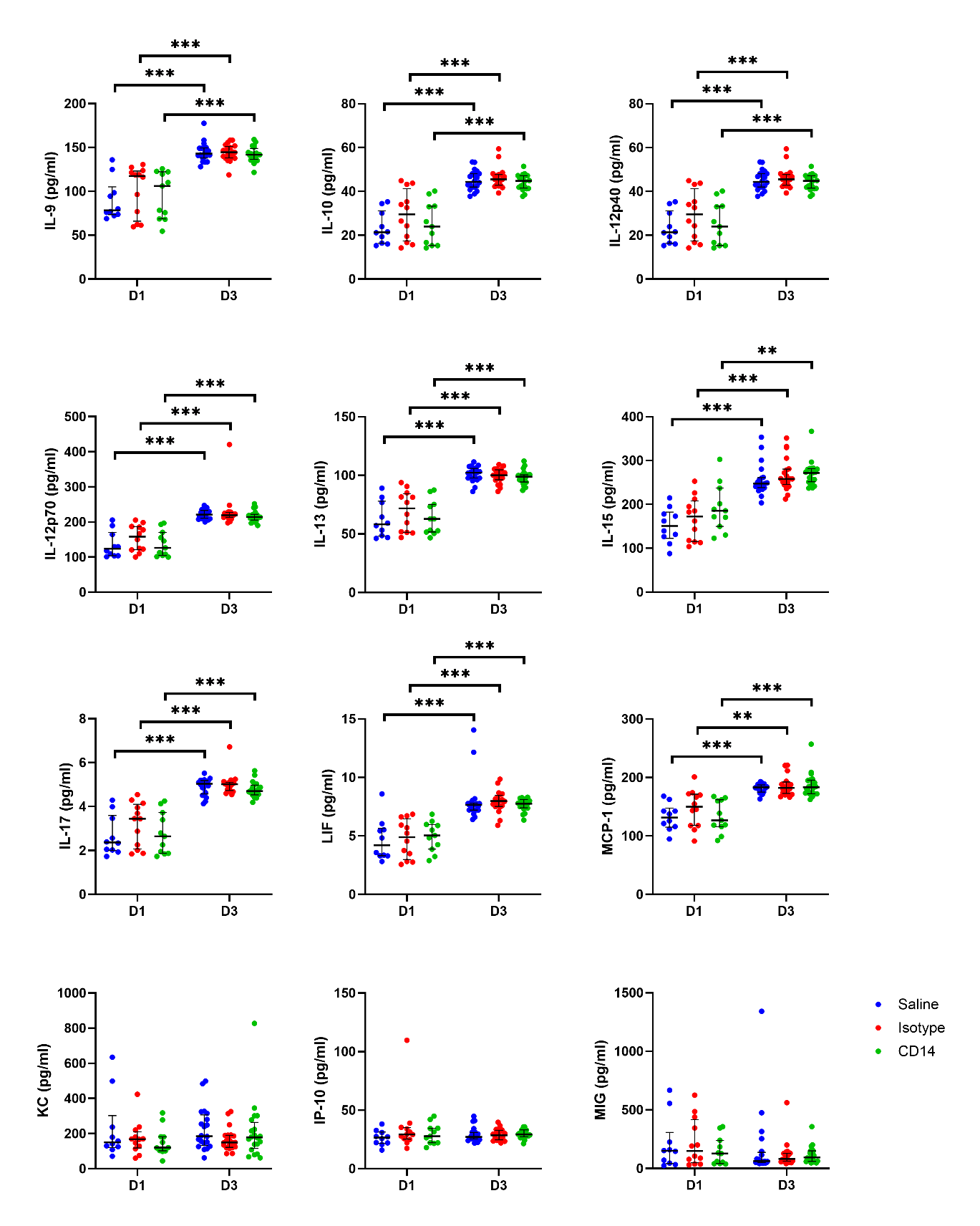


## Supplementary Figure S8. (continued) Circulating Growth Factors following CD14 Blockade at D1 and D3 post-STEMI. Data presented as median (Q1-Q3), with Mann-Whitney U-test conducted between D1 and D3 and Kruskal Wallis test to compare between the three groups at each timepoint. ** p<0.01, *** p<0.001


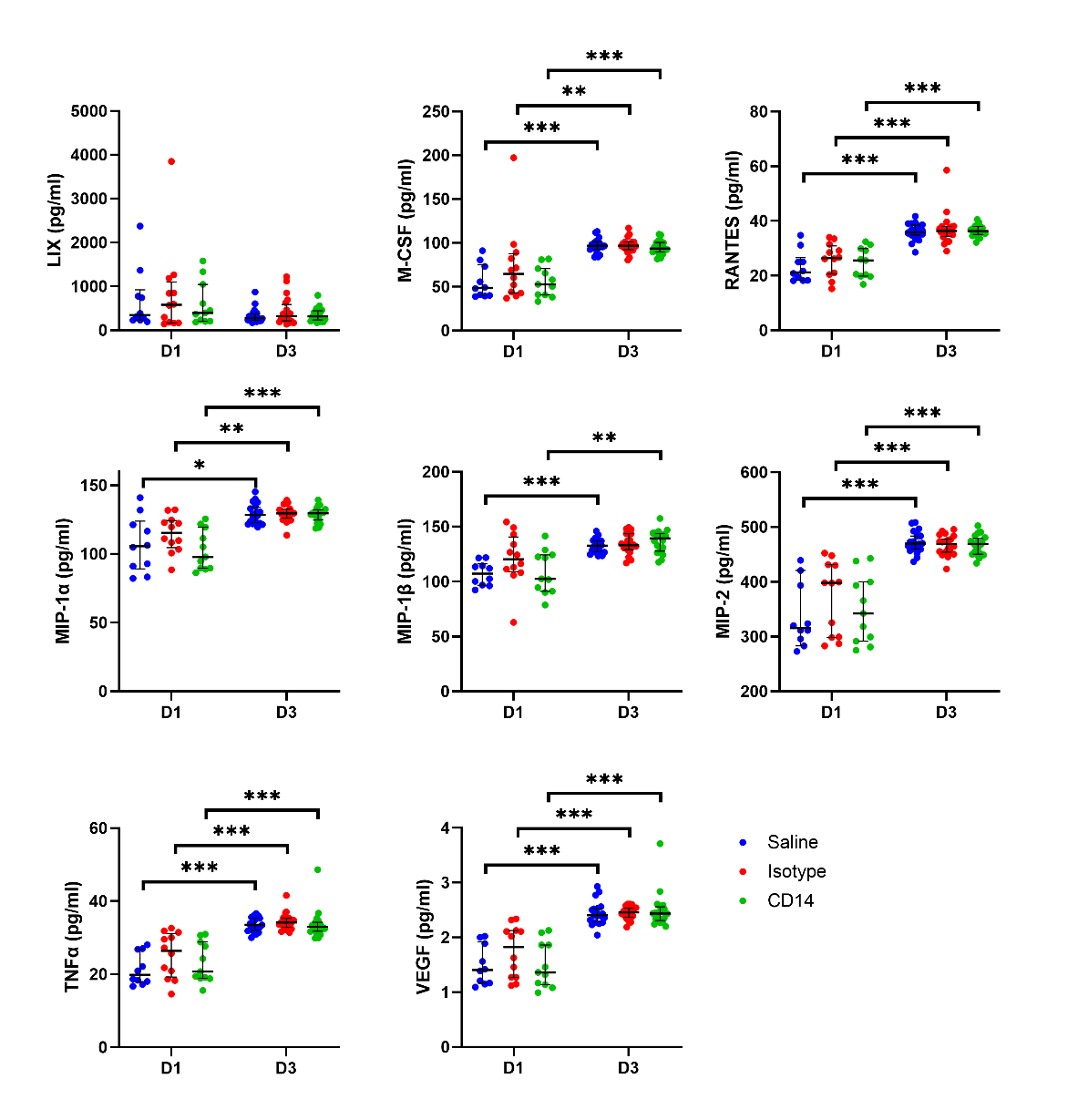


## Supplementary Figure S8. (continued) Circulating Growth Factors following CD14 Blockade at D1 and D3 post-STEMI. Data presented as median (Q1-Q3), with Mann-Whitney U-test conducted between D1 and D3 and Kruskal Wallis test to compare between the three groups at each timepoint. * p<0.05, ** p<0.01, *** p<0.001

##

##
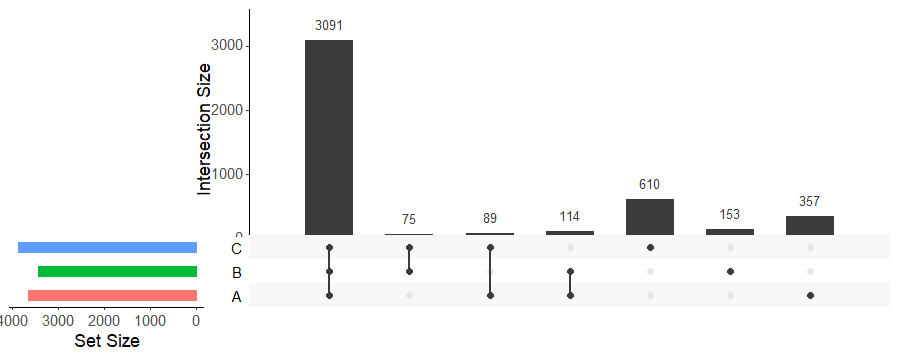

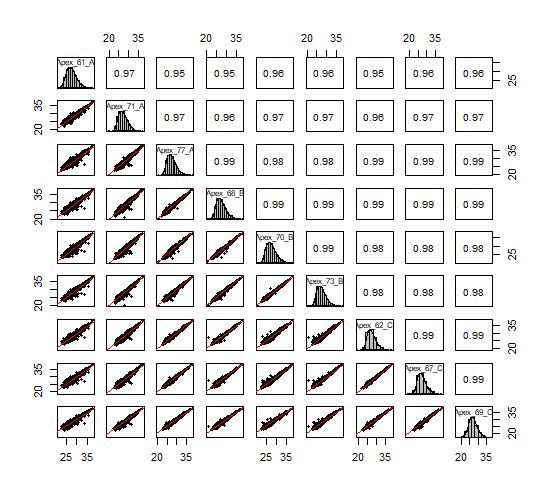


**Saline**

**Isotype**

**Anti-CD14**

**Isotype**

**Anti-CD14**

**Saline**

3651

3433

3865

**F**

**G**

**H**


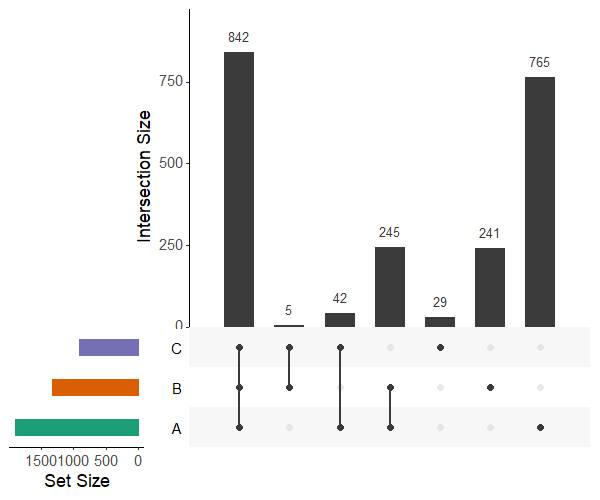

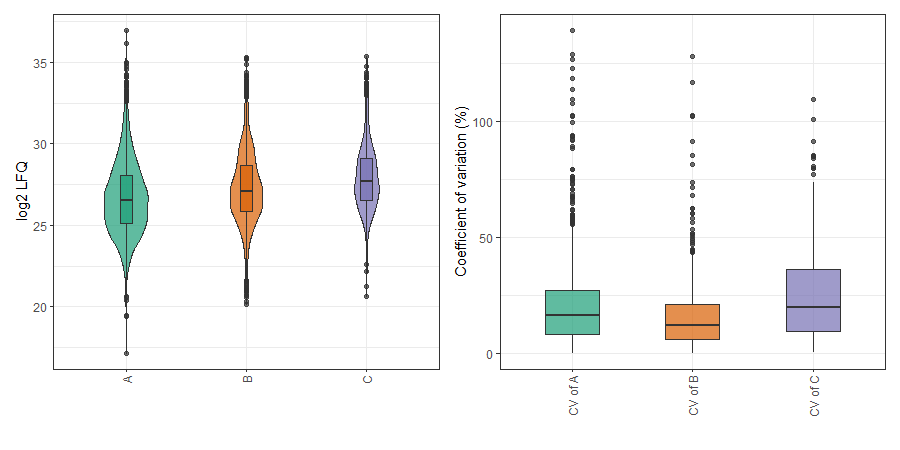

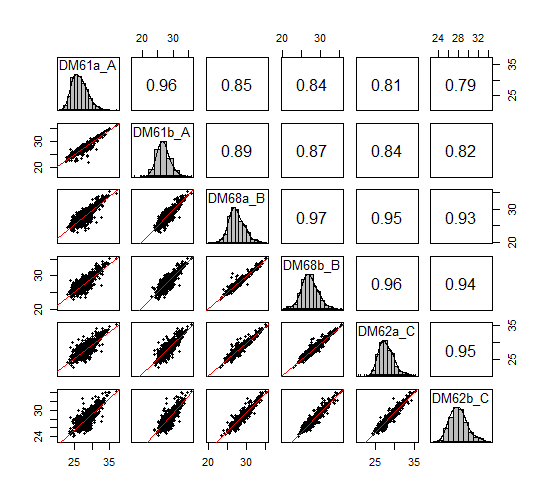

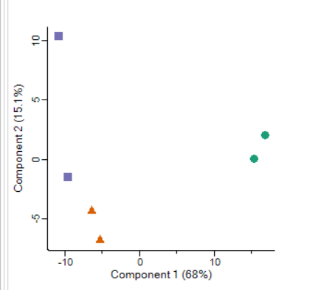


1894

1333

918

**A**

**B**

**C**

**D**

**E**

##
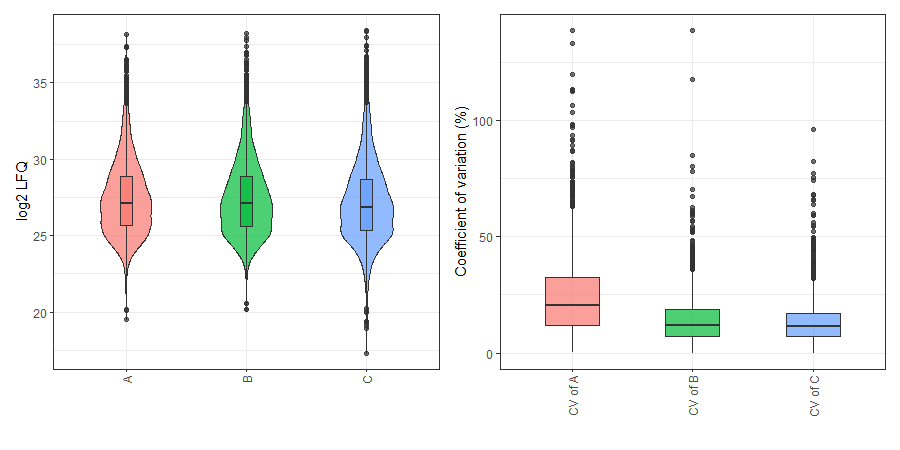

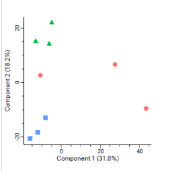


**I**

**J**


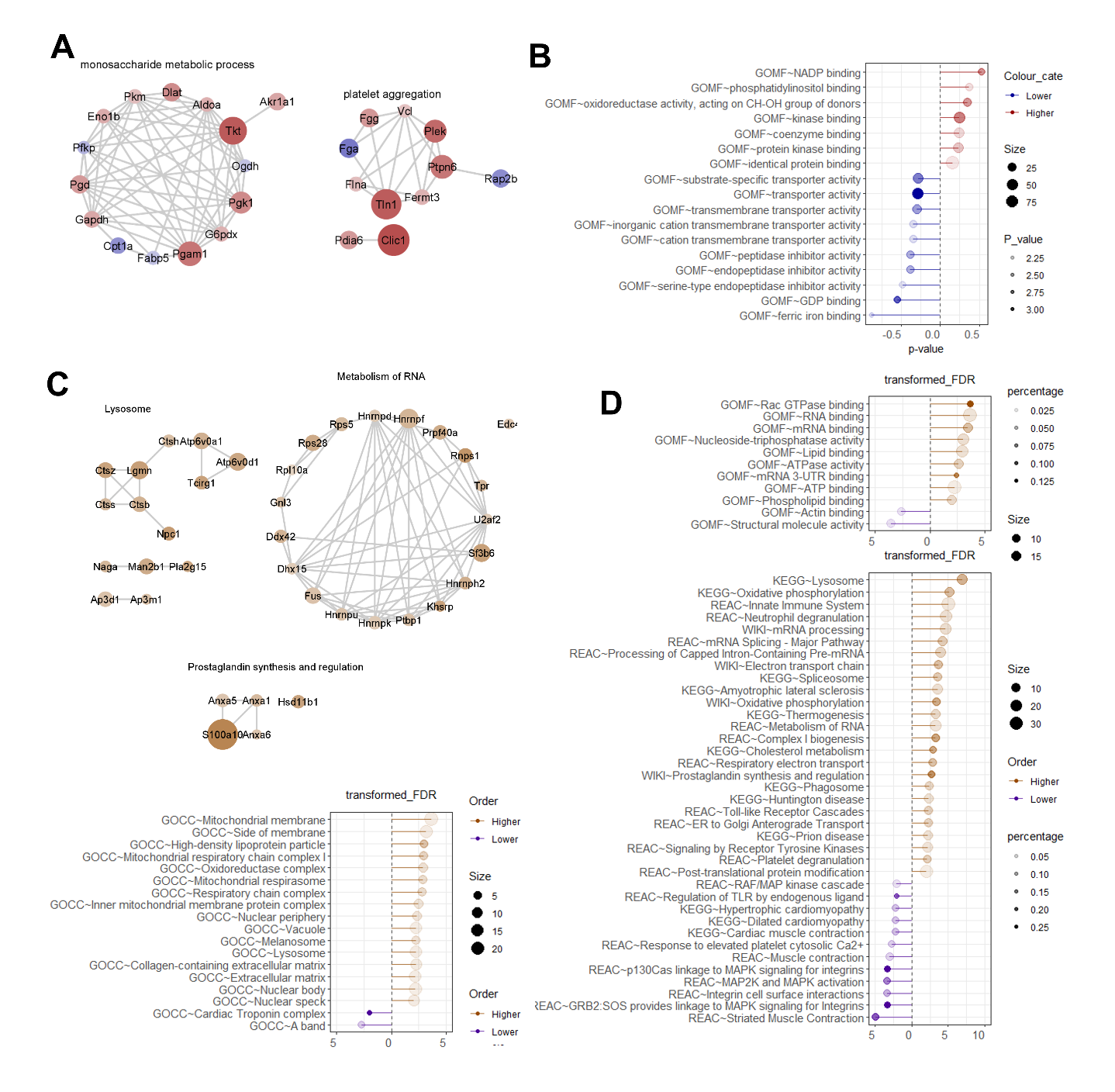


**K**

**L**

**M**

**N**

## Supplementary Figure S9. Proteomics data on macrophages isolated from infarcted LV apex or whole infarcted LV apex tissue at 3 days post-STEMI. (A-B) Proteome depth (quantified proteins for each sample group) from isolated macrophages and low intragroup coefficient of variance (C-D) and high Pearson correlation (E), indicates high sample quality within group as well differences between groups at a whole proteome level. (F-G) Proteome depth (quantified proteins for each sample group) from whole LV infarcted tissue and low intragroup coefficient of variance (H-I) and high Pearson correlation (J), indicates high sample quality within group as well differences between groups at a whole proteome level. These analyses were performed in an unbiased manner while blinded to the following code: A – isotype, B – anti-CD14, C – saline. K) Cytoscape visualization of macrophage-specific key biological processes and important protein drivers. L) 1-dimensional enrichment analysis based on GO annotations of differentially expressed macrophage proteome (GOBP, GOCC, GOMF). M) Cytoscape visualization of whole tissue key biological processes and important protein drivers. N) 1-dimensional enrichment analysis based on GO annotations of differentially expressed whole tissue proteome (GOBP, GOCC, GOMF).


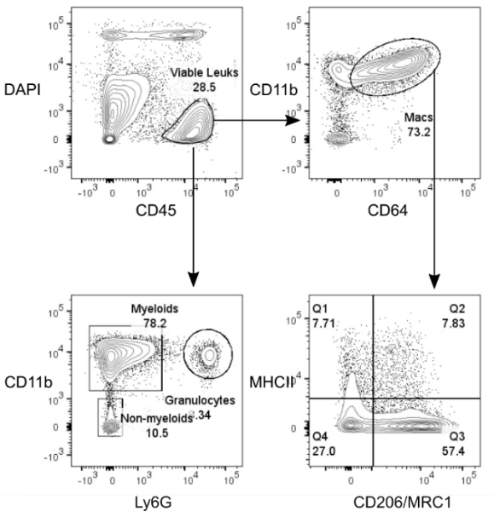


**Supplementary Figure S10. Flow cytometry approach.** Antibodies for CD45, CD11b, CD64, CD14, MHCII, MRC1 and Ly6G were used for sorting macrophages in flow cytometry experiments.


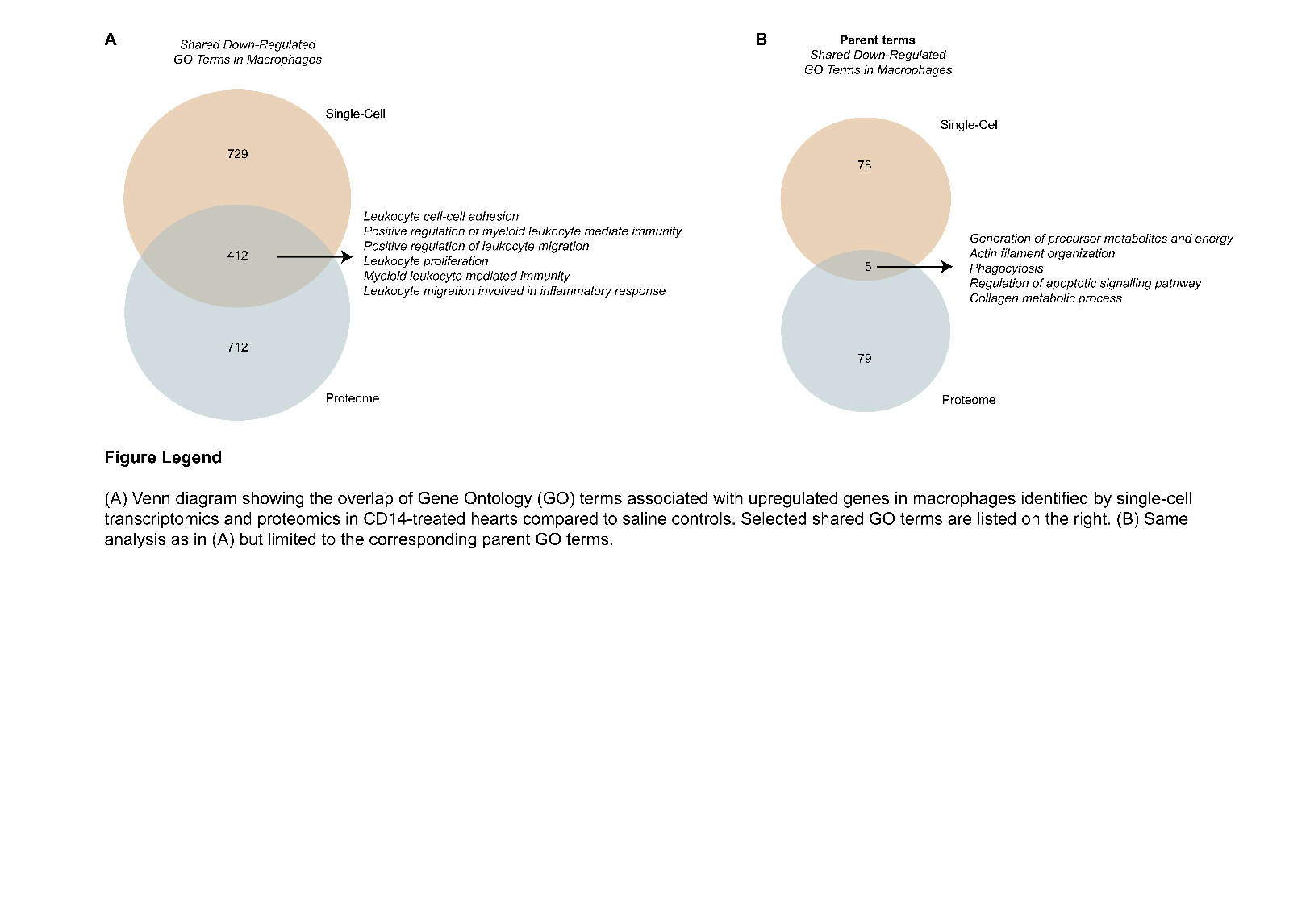


**Supplementary Figure S11. Integrative multi-omic analyses.** (A) Venn diagram showing the overlap of Gene Ontology (GO) terms associated with upregulated genes in macrophages identified by single-cell transcriptomics and proteomics in CD14-treated hearts compared to saline controls. Selected shared GO terms are listed on the right. (B) Same analysis as in (A) but limited to the corresponding parent GO terms.
